# Comparative three-dimensional genome architectures of adipose tissues provide insight into human-specific regulation of metabolic homeostasis

**DOI:** 10.1016/j.jbc.2023.104757

**Published:** 2023-04-27

**Authors:** Pengliang Liu, Diyan Li, Jiaman Zhang, Mengnan He, Dengfeng Gao, Yujie Wang, Yu Lin, Dengke Pan, Penghao Li, Tao Wang, Jing Li, Fanli Kong, Bo Zeng, Lu Lu, Jideng Ma, Keren Long, Guisen Li, Qianzi Tang, Long Jin, Mingzhou Li

**Affiliations:** 1 College of Animal Science and Technology, Sichuan Agricultural University, Chengdu, Sichuan, China; 2 School of Pharmacy, Chengdu University, Chengdu, Sichuan, China; 3 Wildlife Conservation Research Department, Chengdu Research Base of Giant Panda Breeding, Chengdu, Sichuan, China; 4 State Key Laboratory of Agrobiotechnology, College of Biological Sciences, China Agricultural University, Beijing, China; 5 Institute of Organ Transplantation, Sichuan Academy of Medical Sciences & Sichuan Provincial People’s Hospital, Chengdu, Sichuan, China; 6 Jinxin Research Institute for Reproductive Medicine & Genetics, Chengdu Xi’nan Gynecology Hospital, Chengdu, Sichuan, China; 7 Renal Department & Nephrology Institute, Sichuan Provincial People’s Hospital, Chengdu, Sichuan, China

**Keywords:** 3D genome organization, adipose tissue, gene regulation, mammal, thermogenesis, metabolism

## Abstract

Elucidating the regulatory mechanisms of human adipose tissues (ATs) evolution is essential for understanding human-specific metabolic regulation, but the functional importance and evolutionary dynamics of three-dimensional (3D) genome organizations of ATs are not well defined. Here, we compared the 3D genome architectures of anatomically distinct ATs from humans and six representative mammalian models. We recognized evolutionarily conserved and human-specific chromatin conformation in ATs at multiple scales, including compartmentalization, topologically associating domain (TAD), and promoter-enhancer interactions (PEI), which have not been described previously. We found PEI are much more evolutionarily dynamic with respect to compartmentalization and topologically associating domain. Compared to conserved PEIs, human-specific PEIs are enriched for human-specific sequence, and the binding motifs of their potential mediators (transcription factors) are less conserved. Our data also demonstrated that genes involved in the evolutionary dynamics of chromatin organization have weaker transcriptional conservation than those associated with conserved chromatin organization. Furthermore, the genes involved in energy metabolism and the maintenance of metabolic homeostasis are enriched in human-specific chromatin organization, while housekeeping genes, health-related genes, and genetic variations are enriched in evolutionarily conserved compared to human-specific chromatin organization. Finally, we showed extensively divergent human-specific 3D genome organizations among one subcutaneous and three visceral ATs. Together, these findings provide a global overview of 3D genome architecture dynamics between ATs from human and mammalian models and new insights into understanding the regulatory evolution of human ATs.

Adipose tissues (ATs) can range from 5% to 60% of the human body weight (1, 2). They play an essential role in energy storage and mobilization and maintenance of systemic metabolic homeostasis (3). AT dysfunctionality is associated with obesity, type 2 diabetes, dyslipidemia, and nonalcoholic fatty liver (4, 5). Obesity is caused by the excessive accumulation of ATs as a result of energy intake exceeding energy expenditure, which is associated with an increased risk of metabolic disease and the development of numerous cancers (*e.g.*, cancer of the colon, pancreas, liver, and ovary). It even induces adverse outcomes in other diseases (6), such as COVID-19 (3, 7).

ATs in mammals are principally divided into subcutaneous ATs (SATs) and visceral ATs (VATs) based on their anatomical locations. In humans, SAT is located beneath the skin and generally consists of the abdominal, gluteal, and femoral depots. VAT is located in the peritoneal cavity, concentrated in omental and mesenteric depots. The human VAT drains into the portal vein, therefore the free fatty acids and adipokines released from them can be delivered to the liver and influence liver metabolism (8). This increases the metabolic risk of VAT, which is often considered more pernicious than SAT (9). Nevertheless, VAT represents only a small portion (10%–20%) of total fat mass, while SAT accounts for more than 80% in humans (10, 11). SAT plays a critical role in whole-body energy and glucose homeostasis, and preservation of SAT homeostasis can improve systemic metabolic dysfunction in obesity (12, 13). Insufficiency or dysfunction of SATs leads to the lipid spills to other adipose depots (*e.g.*, VATs) and organs (*e.g.*, liver and skeletal muscle), which creates a lipotoxic environment for these organs and subsequently causes an adverse metabolic phenotype (3, 9).

A growing body of evidence has demonstrated that knowledge of genetic diversity across mammals could increase fundamental understanding of human diseases and their unique phenotypes (14, 15, 16, 17). Although storing energy as lipids in ATs is believed to be mechanistically conserved across evolutionary phylogeny (18), physiological differences in ATs have been observed between human and mammalian models (19, 20, 21). Therefore, a better understanding of human AT evolution could contribute to a better understanding of AT-related physiological and pathological mechanisms in humans. In addition, several studies revealed that human-specific three-dimensional (3D) genome organizations could be involved in regulating human-specific gene expression and unique phenotypes (16, 22, 23), suggesting that interspecies comparisons of 3D genomes are valuable for elucidating the genetic basis of the evolution of ATs in humans. Nonetheless, our knowledge of the evolutionary patterns of 3D genome organization between humans and other mammalian ATs remains largely limited. To fill this gap, we systematically compared the 3D genome organizations and transcriptomic characterization of ATs between humans and six other representative mammalian models, including rodents (mice and rats), lagomorphs (rabbits), carnivores (dogs), and artiodactylids (pigs and sheep). We identified the evolutionarily conserved and human-specific chromatin conformation in ATs, ranging from compartmentalization to the topologically associated domain (TAD) and promoter–enhancer interaction (PEI), and investigated the genomic features and functional enrichment of these organizations. These results provide a basis for a deeper understanding of conserved regulatory mechanisms underlying AT functions in mammals and human-specific modulation of biological regulatory networks in ATs.

## Results

### Compendium of the 3D genome organization of ATs across humans and six mammalian models

**Figure 1:**
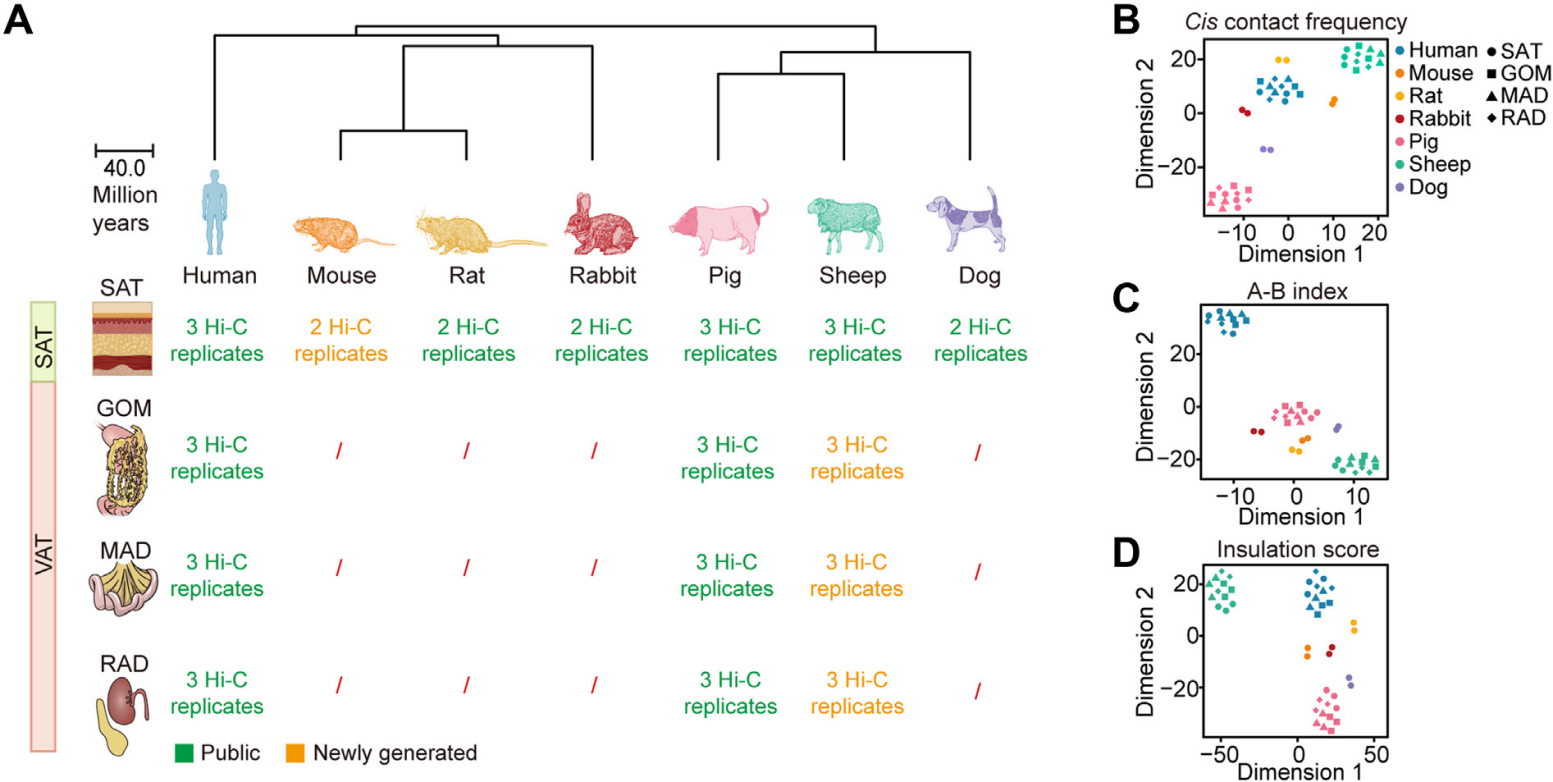
**Overview of Hi-C data**. *A*, Species and ATs were used in this study. ATs were divided into two categories: SAT and VAT (including GOM, MAD, and RAD). SATs were derived from seven mammals, while VATs were derived from three species: humans, pigs, and sheep. *Red slashes* indicate the absence of a Hi-C library in these ATs. GOM, greater omentum; MAD, mesenteric adipose; RAD, retroperitoneal adipose. *B*, t-distributed stochastic neighbor embedding (t-SNE) clustering of samples based on contact frequency between homologous *cis* Hi-C interactions among seven species at 20 Kb resolution. *C* and *D*, t-SNE clustering of samples based on A-B index (*C*) or insulation score (*D*) of 41,770 homologous 20 kb bins between seven mammals. ATs, adipose tissues; GOM, greater omentum; Hi-C, high-throughput chromatin conformation capture; MAD, mesenteric adipose; RAD, retroperitoneal adipose; t-SNE, t-distributed stochastic neighbor embedding; SATs, subcutaneous adipose tissues; VATs, visceral adipose tissues.

To interrogate the 3D genome conformation of ATs in an evolutionary framework, we analyzed the 3D chromatin landscape and transcriptome of SATs collected from humans and other six mammalian models, including rodents (mouse and rats), lagomorphs (rabbits), carnivores (dogs), and artiodactylids (pigs and sheep), and three representative VATs (*i.e.*, greater omentum [GOM], mesenteric adipose [MAD], and retroperitoneal adipose [RAD]) from humans, pigs, and sheep (Table S1). A total of 13.34 billion valid contacts were obtained from *in situ* high-throughput chromatin conformation capture (Hi-C) assays of 44 samples (∼303.20 million [M] contacts per sample), including 33 samples that we published previously (24, 25) (Fig. 1*A* and Table S1). Most (∼69.89%) contacts occurred within chromosomes (Table S1) and showed a distance-dependent interaction frequency decay (Fig. S1*A*), reflecting the reliability of this Hi-C data. Furthermore, the Hi-C maps are highly correlated between biological replicates (median stratum-adjusted correlation coefficient [SCC] = 0.96) (Fig. S1*B*) and more divergent in ATs from different body sites within species (Fig. S1*C*).

We then evaluated the similarity between 3D chromatin organizations across seven mammalian ATs. We first examined the overall similarity among all 44 Hi-C datasets based on the contact frequency of homologous *cis* Hi-C interactions in seven species at a resolution of 20 Kb. The Hi-C samples exhibited a species-dominated clustering pattern (Figs. 1*B* and S2). Furthermore, we compared the chromatin compartmentalization (determined using the A-B index (26)) and local chromatin insulation strength (reflected by insulation score [IS] (27)) based on the homologous genomic regions (comprise ∼29.06% of the human genome) between humans (the ‘reference’ species) and all other six mammalian species. We observed that the samples also mostly separated according to species, with most replicates clustered together (Fig. 1, *C* and *D*). Consistently, the A-B index, IS, and expression of single-copy orthologous genes shared by the seven species repeated this pattern (Fig. S1, *D*–*F*). Altogether, these results demonstrate the divergence of chromatin organizations between species is higher than that between different ATs within species.

### Cross-species comparison identified a cluster of human-specific active compartments that could be associated with maintaining metabolic homeostasis

**Figure 2:**
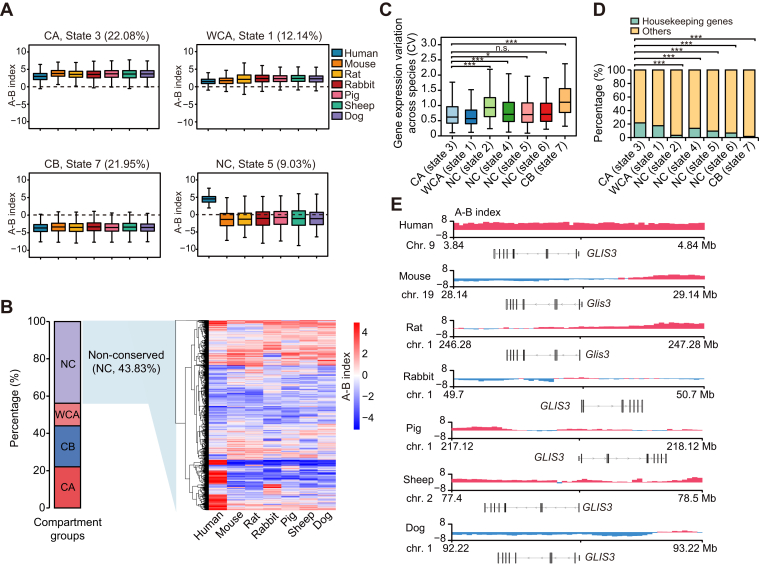
**Evolutionary dynamics of A/B compartment among mammalian SATs**. *A*, four examples of A-B index distributions in Phylo-HMGP states with different patterns (state 3: CA; state 1: WCA; state 7: CB; state 5: NC with human-specific A compartment). *B*, Proportions of each compartment group. The heatmap shows the A-B index for the NC compartment regions. *C*, the expression variation (CV, coefficient of variation across species) of genes located within each compartment state across species. *D*, the fraction of human housekeeping genes within each compartment state. *p*-values were calculated using Fisher’s exact test. ∗∗∗*p* < 0.001 *E*, example showing evolutionary compartment patterns around the *GLIS3* gene among seven mammalian SATs. A-B index are shown and indicate compartment states in each species. SATs, subcutaneous adipose tissues.

To perform high-resolution cross-species 3D genome analyses, we merged the biological replicates for downstream analysis, which reached a resolution of at least 5.2 kb (Fig. S1*G*). This enabled us to explore 3D genome organization at different scales (Fig. S3). We first asked how the A/B compartment changes across mammalian ATs and identified the A/B compartment in seven mammalian SATs using the A-B index algorithm at 20 kb resolution and observed known compartment features in all species (Fig. S4*A*). We next identified the evolutionary patterns of compartment status in homologous genomic regions across seven mammalian species using a continuous-trait probabilistic model (28) (see “Experimental procedures”). We obtained seven evolutionary states of compartment and classified them into four groups: (1) conserved A compartments (CA group, state 3); (2) conserved B compartments (CB group, state 7); (3) weakly conserved A compartments (WCA group, state 1); and (4) non-conserved compartments (NC group, state 2, 4, 5 and 6) (weakly conserved B compartments [WCB] group have not been observed) (Fig. S4*B* and Table S2). In the CA group, more than 90% of the regions had compartment A status across all seven species, while that proportion was 70% to 90% in the WCA group. We similarly assigned states to the CB or WCB. The remaining states were assigned to the NC group. The representative A-B index patterns of the groups are shown in Figure 2*A*.

We found that more than half of the homologous regions (∼56.17%) had conserved or weakly conserved compartment status across the seven mammal species, with the remaining 43.83% assigned to the NC group (Fig. 2*B*). Among the NC states, state five was identified as human-specific (HS) A compartment (75.46 Mb, ∼9.03%). We further explored the features of distinct compartment states and noticed that the majority of the 20-kb regions in NC compartments exhibited relatively “intermediate” status with low absolute A-B values compared to conserved or weakly conserved compartments (Fig. S4*C*). We next examined the genes embedded in these regions. We found that single-copy orthologous genes embedded in most NC compartments showed significantly decreased transcriptional stability between species compared to those embedded in the CA and WCA compartments (Fig. 2*C*). Notably, the genes embedded in CB compartments showed low expression stability, most likely due to the very low expression levels among these genes across the seven species (∼50% of these genes have a median TPM<0.5 in the seven species). This finding indicates a functional link between evolutionary dynamic compartments and altered expression in mammalian SATs. Housekeeping genes help maintain basal cellular functions (29). We found that the human housekeeping genes (29) were more enriched in the CA and WCA states compared to other compartment states (Fig. 2*D*). This suggests these genes favor active compartments and are prone to maintaining their active compartment status across species. Functional enrichment analysis suggested that genes embedded in CA regions were primarily involved in basic cellular processes (such as nucleocytoplasmic transport and cytoplasmic translation) and response to DNA damage (Fig. S5 and Table S3). The genes embedded in WCA regions were primarily enriched in categories related to disease and immunity while those in CB regions were mainly associated with neurotransmission and chemosensory signaling, and those present in NC regions were involved in the development and biological processes related to AT functions (such as catecholamine metabolic process, regulation of phospholipase activity) (Fig. S5 and Table S3). These observations underline divergences in AT function among species.

We further focus on the potential biological functions of the HS A compartment state (*i.e.*, state 5, embedded with 199 genes, Table S4). We found that genes related to glucose and energy homeostasis (*e.g.*, “regulation of activin receptor signaling pathway”) were enriched in this state (Fig. S5). Notably, “hydrogen peroxide metabolic process” is the most enriched term (*p* < 10^−4^) (Fig. S5), which has been linked to maintaining metabolic homeostasis. Inhibiting this process in AT triggers oxidative stress and the deterioration of insulin sensitivity, which can eventually lead to metabolic disorders (30, 31). Indeed, genes known to play a role in metabolic homeostasis were observed in HS active compartments. One notable case is *GLIS3*, an essential transcription factor involved in regulating glucose metabolism (32, 33). Mutations in this gene can lead to neonatal diabetes in humans (34), and showed a more active compartment status in humans compared to other mammals (Figs. 2*E* and S4*D*). This suggests that HS active compartments could have potentially beneficial effects on metabolic homeostasis in human SAT.

### HS TADs in SATs

**Figure 3:**
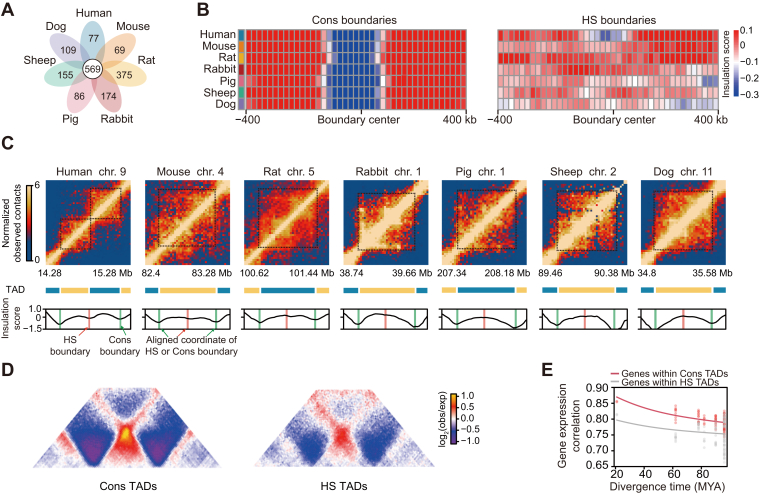
**Identification of human-specific TADs during the evolution of mammalian SATs.***A*, flower plot showing the species-conserved and species-specific TAD boundary numbers. *B*, average insulation score profiles in 400-kb regions centered on species-conserved and human-specific TAD boundaries at 20 kb resolution. Cons boundaries, species-conserved boundaries; HS boundaries, human-specific boundaries. *C*, representative Hi-C contact maps and insulation score profiles around HS TADs in seven mammalian SATs at 20-kb resolution. The HS and Cons TAD boundaries are highlighted with red and green bars, respectively, in the insulation score profiles in humans. Their genomic coordinates on other species were obtained using the UCSC liftover tool and are highlighted with red (HS boundary) or green (Cons boundary) bars. The dashed lines in the Hi-C contact maps indicate TADs. *D*, aggregate Hi-C maps showing the average intra- and inter-TAD contact frequency of the species-conserved and human-specific TADs in human SAT. Cons TADs, species-conserved TADs; HS TADs, human-specific TADs. *E*, pairwise Spearman’s correlation coefficients of expression levels between species were plotted against the evolutionary distance of single-copy orthologous genes located within Cons TADs (*n* = 278) or HS TADs (*n* = 411). The lines correspond to nonlinear regression trends using nonlinear least squares. HS, human specific; SATs, subcutaneous adipose tissues; TAD, topologically associating domain.

TADs are known as highly self-interacting fundamental units of 3D chromatin organization (35). To characterize the evolutionary dynamics of TADs in SAT, we identified ∼3546 TADs (range from 2979 to 4411, with ∼500 kb median length) in each mammalian SAT (Fig. S6, *A* and *B*). Supporting the extensive conservation of TAD structures among mammals (35, 36), we found 569 species-conserved (Cons) TAD boundaries and only 77 HS TAD boundaries (Figs. 3, *A* and *B*, S6*C* and Table S5). We next compared the features between Cons and HS TAD boundaries. We found that Cons boundaries are more preferentially located in evolutionarily conserved compartments than HS boundaries (41.83% vs. 29.87%, *p* = 0.048, Fisher’s exact test) (Fig. S6*D*). We also observed housekeeping genes are more concentrated in Cons boundaries than in HS boundaries (Fig. S6*E*), which is similar to previous findings regarding the evolution of the human brain (16).

Based on these boundaries, we defined 124 Cons TAD (both TAD boundaries are Cons boundaries) and 143 HS TADs (at least one side of the TAD boundary is HS boundary) in the human genome. Representative HS TAD is illustrated in Figure 3*C*. The HS TADs (median size of 480 kb) are generally shorter than Cons TADs (median size of 600 kb) (Fig. S6*F*), which is consistent with a previous study that observed large conserved TADs by comparing mammals (16, 37). We also found that the Cons TADs exhibited stronger intra-TAD interactions than species-specific TADs (Figs. 3*D* and S6*G*). Notably, the HS TADs have higher gene density than Cons TADs (*p* = 0.011, Wilcoxon rank-sum test) (Fig. S6*H*), suggesting a functional role of HS TADs. We next focus on 429 and 803 genes within Cons and HS TADs, respectively. We observed the expression changes of single-copy orthologous genes within HS TADs are more dramatic across mammalian SATs than within Cons TADs (Fig. 3*E*). This indicates that TADs that are rearranged during human SAT evolution also contribute to changes in gene expression. Functional enrichment showed that genes within HS TADs (Table S4) were enriched in categories related to the immune system, chromosome organization, development, and morphogenesis (Fig. S6*I* and Table S6). Moreover, we also observed an enrichment of glucose and energy metabolic-related categories (*e.g.*, “amylin receptor signaling pathway”) in HS TADs (Fig. S6*I*).

### Comparing species-conserved and HS PEIs

**Figure 4:**
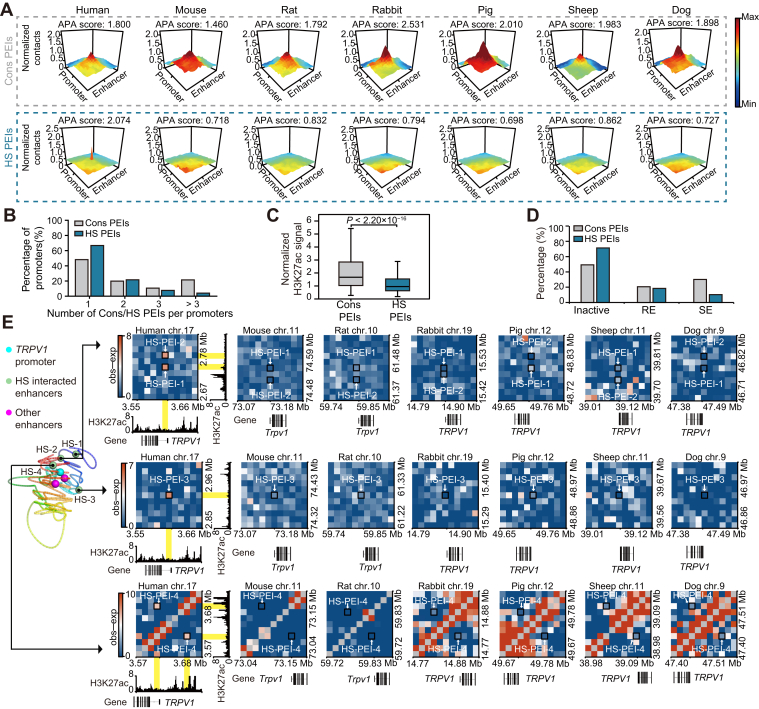
**The species-conserved and human-specific PEIs across mammalian SATs.***A*, normalized Hi-C signals around species-conserved and human-specific PEIs in each mammalian SAT at 10 kb resolution. APA scores were obtained from Juicer tools. Cons PEIs, species-conserved PEIs; HS PEIs, human-specific PEIs. *B*, bar plots showing the percentage of promoters forming one to more than three Cons or HS PEIs. *C*, normalized H3K27ac signals in the enhancer regions of Cons and HS PEIs in human SAT. *p* values were calculated by Wilcoxon rank-sum test. *D*, percentage of regular enhancers (REs) and SEs in Cons and HS PEIs. *E*, example of HS PEIs of *TRPV1* gene. *Left*: 3D model of the promoter (*blue sphere*) and enhancers (*green*: human-specific; purple: others). *Right*: Local contact maps around 4 HS PEIs (indicated by *black square*) associated with *TRPV1* gene at 10 kb resolution. PEI anchors are highlighted in *yellow*. AT, adipose tissues; HS, human specific; PEI, promoter-enhancer interaction; SATs, subcutaneous adipose tissues; TAD, topologically associating domain.

Physical interaction between the enhancer and its remote target promoter is essential in mammalian transcriptional control (38). To explore the evolutionary dynamics of PEI organization in SATs, we identified ∼43,273 PEIs (range from 22,269–66,464) in each mammalian SAT using the PSYCHIC algorithm (39) at 10 kb resolution. Consistent with the enhancers providing an additive effect on target gene transcription (40, 41, 42), gene expression was positively correlated with the number of their interacted enhancers (Fig. S7*A*). We next separately aligned 10,213 PEIs of human SATs to the genomes of other six mammals to compare PEI organizations and observed more HS PEIs (21.68%, or 2214 of 10,213) than Cons PEIs (8.79%, or 898 of 10,213) (Fig. S7*B* and Table S7). These PEIs were confirmed by aggregating peak analysis (Figs. 4*A* and S8). Interestingly, we found Cons PEIs preferentially regulated the same promoter (*i.e.*, many to one) compared to HS PEIs (Fig. 4*B*). This is likely due to multiple conserved enhancers, which can better confer robustness on gene expression during evolution since one or more redundant enhancers compensate for the loss.

To investigate the enhancer activity of our identified Cons and HS PEIs, we integrated them with public human SAT H3K27ac ChIP-seq signals (25), which reflect enhancer activity. Remarkably, the enhancer regions of Cons PEIs exhibited a stronger H3K27ac signal than HS PEIs (Fig. 4*C*). Additionally, Cons PEIs are significantly more enriched for super-enhancers (SEs) than HS PEIs (30.18% vs. 10.25%, *p* < 2.20 × 10^−16^, Fisher’s exact test), while no such enrichment was detected for regular-enhancers (REs) (20.60% vs. 18.38%, *p* = 0.16, Fisher’s exact test) (Fig. 4*D*). This finding suggests that the interactions between human SE regions and their target genes tend to be conserved during SAT evolution.

To gain functional insights into these PEIs, we examined 378 and 1463 genes regulated by Cons and HS PEIs, respectively. Compared to Cons PEIs associated genes, genes regulated by HS PEIs (Table S4) tend to be slightly upregulated in humans compared to other mammalian species (*p* = 0.069, Student’s *t* test) (Fig. S7*C*). We found genes related to lipid metabolism categories (such as ‘positive regulation of fatty acid oxidation’, ‘unsaturated fatty acid metabolic process’, and ‘regulation of phosphatidylinositol 3-kinase signaling’), were overrepresented in HS PEI-regulated genes compared to those of Cons PEIs (Fig. S7*D* and Table S8). For example, *TRPV1*, which is associated with energy and lipid metabolism (43), was formed by four HS PEIs and concomitant with the upregulation of gene expression in human SAT compared to other species (Figs. 4*E* and S7*E*). To test whether the identified four HS PEIs of the *TRPV1* gene were also present in adipocytes, we examined their contact frequency in human and mouse adipocytes using previously published Hi-C datasets (44, 45). As expected, the clear Hi-C signals of these four HS PEIs were almost entirely detected in human adipocytes (Fig. S7*F*).

Genes involved in diseases (*e.g.*, “AGE-RAGE signaling pathway in diabetic complications” and “Lipid and atherosclerosis”) were enriched in Cons PEIs (Fig. S7*D*), indicating that these conserved 3D chromatin structures are important for the health of organisms. To further understand the relevance of these PEIs and human health, we collected four GWAS datasets related to AT-related diseases from a previously described GWAS set (46), including “diabetes mellitus” (combined three GWAS sets, a total of 5349 significant SNPs, *p* < 5 × 10^−8^) and “disorders of lipoprotein metabolism and other lipidemias” (2499 significant SNPs, *p* < 5 × 10^−8^) traits (Table S9). We found that the Cons PEIs enhancers are significantly more enriched for diabetic SNPs than HS PEIs (*p* = 0.043, Fisher’s exact test) (Fig. S7*G*). SNPs associated with “disorders of lipoprotein metabolism and other lipidemias” displayed a similar trend, although it was not significant (Fig. S7*G*). These findings suggest that regulatory structures associated with health risks tend to be conserved between humans and other mammalian SATs.

Notably, genes related to thermogenesis and temperature regulation (*e.g.*, “regulation of cold-induced thermogenesis,” “TGF-β signaling pathway,” and “brown fat cell differentiation”) were also preferentially enriched in Cons PEIs (Fig. S7*D*). To confirm this observation, we examined the enrichment of Cons, and HS PEIs regulated genes in two previously reported thermogenesis-related adipocyte subpopulations in mice (20). The Cons PEIs regulated genes were more enriched in these two subpopulations than those of HS PEIs (Fig. S7*H*), implying that thermogenic regulation of ATs is often conserved at the PEI level.

### Dynamic binding of transcription factors between conserved and HS PEIs

**Figure 5:**
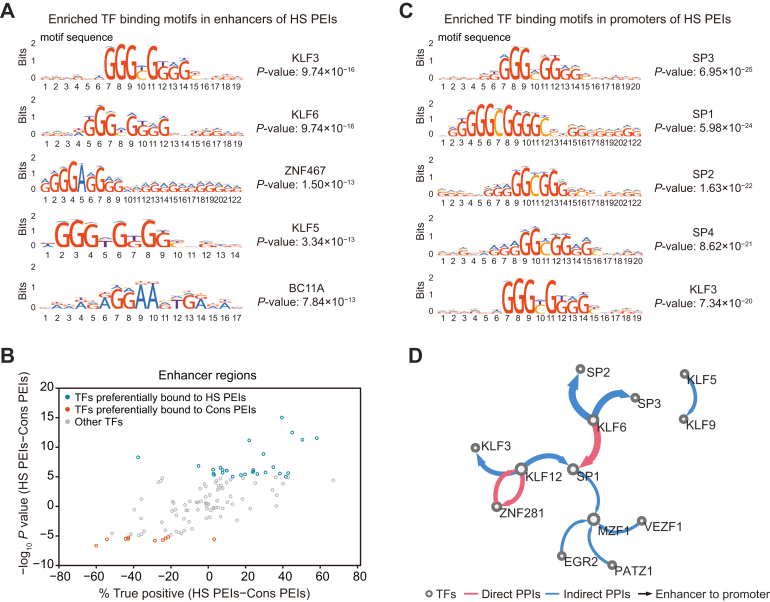
**The transcription factor motifs associated with human-specific PEIs**. *A*, top five enriched TF binding motifs within the enhancer regions of HS PEIs. *B*, the enrichment changes for TFs at the enhancer regions of HS PEIs compared to that of Cons PEIs. *C*, top five enriched TF binding motifs within the promoter regions of HS PEIs. *D*, TF interaction network shows the candidate TF pairs associated with PEI formation and HS PEIs binding preference. Each node represents 1 TF. Each edge indicates a predicted physical interaction between 2 TFs. The *blue* and *red edges* represent indirect and direct PPIs in the STRING database, respectively. The width of the edge indicates the interaction frequency, and the direction of the arrow is from enhancer to promoter. The size of the circle matches the node degree. HS, human specific; PEI, promoter-enhancer interaction; PPI, protein–protein interaction; TF, transcription factor.

Transcription factors (TFs) are usually bound to enhancers and mediate communication with their target promoters (38, 47). To this end, we conducted TF motif analysis for the open chromatin regions in enhancers of HS and Cons PEIs using the public ATAC-seq profile for human ATs (21). We observed distinct enrichment of TF motifs between HS and Cons PEIs (Figs. 5*A* and S9*A*). The top five enriched motifs in HS PEIs enhancers are primarily involved in adipogenesis, including three KLF factors (KLF3, KLF6, and KLF5) (48) and ZNF467 (49) (Fig. 5*A*). In contrast, the top five enriched motifs in Cons PEIs enhancers are related to controlling immune and inflammatory response (IRF3 (50) and ETV5 (51)), adipogenesis (EGR2 (52) and ETS2 (53)), and angiogenesis (VEZF1 (54)) (Fig. S9*A*). We further defined 28 and 10 enhancer-TFs whose binding displayed HS or Cons PEIs preference (Fig. 5*B* and Table S10, see “Experimental procedures”), respectively. Notably, ∼25% (7 of 28) of TFs with HS PEIs-binding preference belong to KLF families, suggesting that KLF factors could play an important role in the human-specific regulation of SAT functions. Furthermore, we examined TFs that bind to promoters of PEIs and found that the most enriched TF motifs in HS PEIs promoters are related to the Sp family (SP3, SP1, SP2, SP4) (Fig. 5*C*), which are distinct from the enhancer-TFs observed above.

We next predicted pairwise TFs that are potentially involved in PEI formation (*i.e.*, 1 TF binds to the promoter and the other to an enhancer) and displayed a significant preference for HS PEIs compared to Cons PEIs (see “Experimental procedures”). We identified a total of 12 TF pairs that preferentially bind to HS PEIs (Fig. 5*D* and Table S11). Of these, three TF pairs overlap with direct protein–protein interactions (PPIs) in the STRING database (55), including KLF6-SP1, ZNF281-KLF12, and KLF12-ZNF281 (directions from enhancer to promoter). Finally, we examined the sequence conservation of the binding sites of these 12 TF pairs in HS and Cons PEIs, respectively. We found 10 promoter-TFs whose binding sites have significantly lower sequence conservation (determined by phastCons score) in HS PEIs than in Cons PEIs (Fig. S9*B*), indicating that the HS PEIs that preferred binding with these promoter-TFs could be sequence-driven. This highlights that the conservation of the TF binding sites in promoters is important for future studies, which could play a role in mammalian evolution by affecting PEI formation or loss. In contrast, the most binding sites of enhancer-TFs showed comparable sequence conservation between HS and Cons PEIs, with only 2 TFs whose binding sites exhibited significantly lower phastCons scores in HS PEIs (Fig. S9*B*). This observation could partly be due to the fact that enhancer-TF binding sites were more commonly located in intragenic regions in HS PEIs compared to those in Cons PEIs (Fig. S9*C*).

### HS 3D chromatin structure changes linked to genomic sequence changes

**Figure 6:**
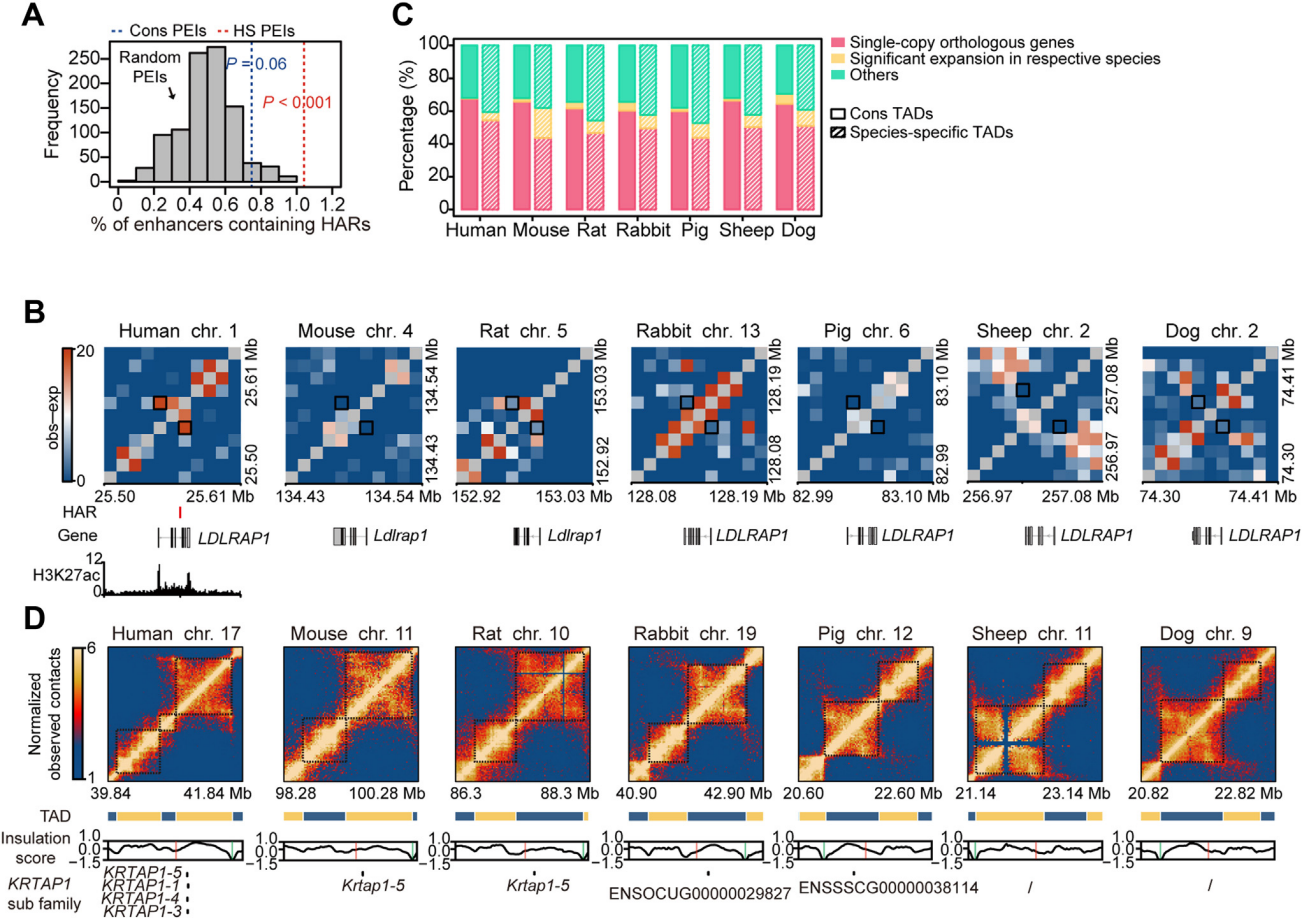
**The genomic basis of human-specific chromatin structural changes.***A*, percentage of enhancers containing at least one HAR for Cons and HS PEIs. Histogram showing the frequency of percentage of random PEIs containing at least one HAR in human SAT. *p* values were calculated by permutation test. *B*, local contact maps of an HS PEI example (indicated by *black square*) associated with HAR in human SAT. HAR is highlighted with a *red bar* in human SAT. *C*, the proportion of single-copy orthologous genes and the genes that significantly expanded in each mammal's respective species within Cons or species-specific TADs. *D*, example of contact matrix and insulation score profiles around *KRTAP1* gene family at 20 kb resolution. The HS boundary is highlighted with a *red bar*, and the Cons boundary is highlighted with a *green bar*. *Black slashes* indicate the absence of the *KRTAP1* gene family members in sheep and dogs. HARs, human accelerated regions; HS, human specific; PEI, promoter-enhancer interaction; SATs, subcutaneous adipose tissues; TAD, topologically associating domain.

We next examined the genomic basis of HS 3D chromatin organization (56). Human accelerated regions (HARs; mammalian/non-human species conserved regions that are accelerated in humans) can serve as enhancers to implicate the evolution of uniquely human traits (57, 58). We assessed whether HARs are enriched in HS PEIs enhancers. Analysis of previously reported HARs set (*n* = 2737) (57) revealed that enhancers of HS PEIs are significantly enriched for HARs compared to randomly selected enhancers (*p* < 0.001, permutation test), while this enrichment was not observed in Cons PEIs enhancers (*p* = 0.06, permutation test) (Fig. 6*A*). This indicates that HAR could serve as an enhancer and involve human SAT evolution through spatial chromatin interaction. A remarkable case showing one HAR (HAR_Merge50–00012) overlaps with an active enhancer of HS PEIs and targets the *LDLRAP1* promoter (Fig. 6*B*), which plays an important role in removing low-density lipoprotein cholesterol from the circulatory system; the mutation of *LDLRAP1* causes autosomal recessive hypercholesterolemia in humans (59).

Additionally, at the TAD scale, we found the genes whose family underwent significant expansion in humans are more enriched in HS TADs than Cons TADs (4.98% vs. 0.47%, *p* = 7.89 × 10^−6^, Fisher’s exact test). In contrast, single-copy orthologous genes were significantly depleted in HS TADs (51.06% vs. 64.57%, *p* = 0.018, Fisher’s exact test) (Fig. 6*C*). Similar patterns were observed in other mammals (Fig. 6*C*). These findings suggest that the expansion of the gene families could be associated with the evolutionary rearrangement of TADs. One example of these HS TADs can be found around the *KRTAP1* gene family in the human genome, whose expansionary members reside close to an HS boundary (Fig. 6*D*).

### 3D genome evolutionary dynamics differ between anatomical distinct adipose tissues

**Figure 7:**
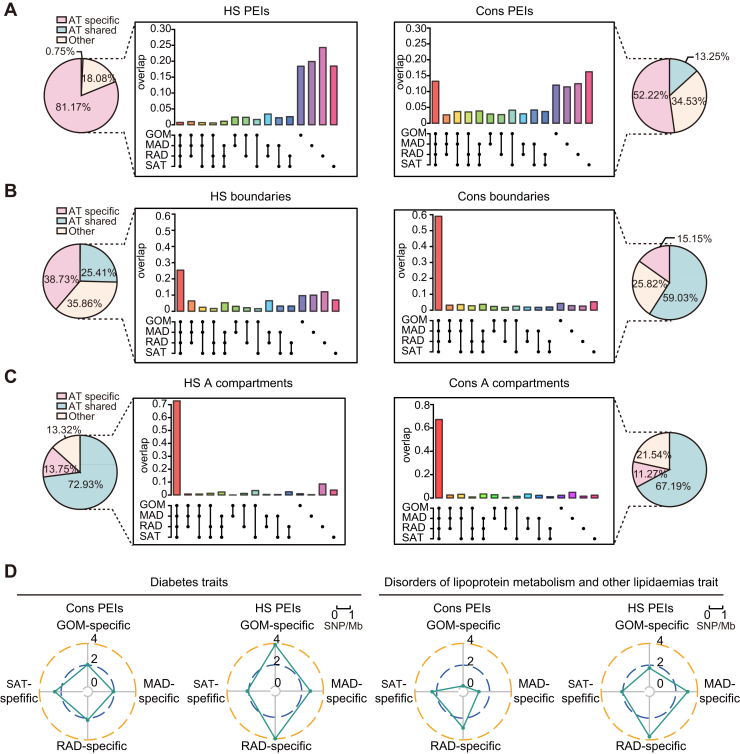
**Human-specific 3D chromatin structures of each adipose tissue varied more than species-conserved structures**. *A* and *B*, Overlap of identified HS and Cons PEIs (A), TADs (B), in each of four ATs. The bars are a numerical sum of the human-specific (*left panel*) or conserved (*right panel*) 3D chromatin structures identified in all four ATs. The pie charts show the portions of AT shared or specific 3D chromatin structures. *C*, Overlap of identified HS A compartments (*left panel*) and Cons A compartments (*right panel*) in four ATs. *D*, Radar plots show SNP density linked to “diabetes” and “disorders of lipoprotein metabolism and other lipidaemias” traits in the enhancer regions of AT-specific Cons and HS PEIs. Lines connecting the density values to the origin of the plot have been added to improve visualization. HS, human specific; PEI, promoter-enhancer interaction; TAD, topologically associating domain.

It has been posited that ATs are usually distributed throughout the body in several discrete anatomical locations in mammals, which differ in their morphologies, structural organization, and physiological functions. These structures are even treated as separate “miniorgans” by some scholars (2, 18). As such, in order to fully appreciate the evolution of human ATs, it is necessary to also evaluate specific AT types. To advance our understanding of 3D genomic evolution in distant AT types, we first identified Cons and HS 3D chromatin structures (including compartments, TADs, and PEIs) across three species (humans, pigs, and sheep) in four ATs (SAT, GOM, MAD, and RAD) using the same approach as described in earlier comparative analysis of seven mammalian SATs. (Fig. S10, *A*–*C* and Tables S12–S14). Representative examples of HS and Cons chromatin structures are shown in Fig. S11. We then investigated the maintenance of HS or Cons 3D genome structures across anatomical distinct ATs by investigating how often they aligned to a similar HS or Cons chromatin structure in another AT. The chromatin structures consistently identified as HS/Cons in all four ATs were defined as AT-shared HS/Cons chromatin structures. In contrast, chromatin structures identified as HS/Cons in only one AT were defined as AT-specific HS/Cons chromatin structures. We found that the HS PEIs were shared less among four AT types than Cons PEIs (0.75% vs. 13.25%, *p* < 2.20 × 10^−16^, Fisher’s exact test) (Fig. 7*A*). A similar observation was found for TADs, where 25.41% of HS boundaries were AT-shared compared to 59.03% of Cons boundaries (*p* < 2.20 × 10^−16^, Fisher’s exact test) (Fig. 7*B*). In contrast, at the compartment level, most (72.93%) HS A compartments (including state five for SAT, five for GOM, six for MAD, and four for RAD) (Fig. S10*A*) were shared among ATs, which was comparable with conserved A or B compartments (67.19% of conserved A compartments and 74.80% of conserved B compartments were shared among ATs) (Figs. 7*C* and S10*D*). This suggests that these HS A compartments may have an important role in human ATs. Altogether, these findings suggest that the HS chromatin structures (especially for TADs and PEIs) between species within one AT type are relatively rare and not maintained as HS chromatin structures in other AT types.

We then explored the possible function of AT-specific HS chromatin structures at the PEI and TAD levels, except for the compartments, since limited AT-specific HS active compartments were detected (Fig. 7*C*). Interestingly, we found that categories related to immunity and inflammation are preferentially enriched in the HS TADs and PEIs specifically occurred in VATs, particularly in RAD and MAD, compared to those that specifically occurred in SAT (Fig. S12, Tables S15 and S16). There is growing evidence that VATs have more immune and inflammatory features than SATs (60). The discordant 3D genome evolution among one human SAT and three VATs may provide clues about the molecular basis underlying inflammatory and immune differences between SAT and VAT.

Notably, we found that genes related to metabolic diseases (*e.g.*, “diabetic cardiomyopathy”) were enriched in HS PEIs that specifically occurred in VATs (Fig. S12*B*), implying that these structures may carry higher disease risks than SAT-specific HS PEIs. To further confirm this hypothesis, we estimated the enrichment of the above-described disease-related SNPs (Table S9) in the enhancer regions of AT-specific HS PEIs. We found that diabetes SNPs are depleted in SAT-specific HS PEIs compared to the specific HS PEIs for each of other three VATs (*p* < 4.39 × 10^−3^, Fisher’s exact test) (Fig. 7*D*). A similar result was observed for SNPs related to “disorders of lipoprotein metabolism and other lipidemias.” SAT-specific HS PEIs showed significantly lower proportions than RAD- and MAD-specific HS PEIs (*p* < 4.72 × 10^−4^, Fisher’s exact test), but were comparable to GOM-specific HS PEIs (*p* = 0.22, Fisher’s exact test) (Fig. 7*D*). However, these patterns were not observed when we investigated SNP enrichment in enhancer regions of AT-specific Cons PEIs. Taken together, HS 3D chromatin structures are widely discordant among one SAT and three VATs, which is correlated with the differences in functional properties between SAT and VAT.

## Discussion

While previous studies reported anatomical, physiological, and morphological differences between ATs across different species (61, 62), little is still currently known about their properties at the molecular levels. In this study, we performed a 3D genome comparison between ATs in humans and other six mammalian species, uncovering evolutionarily conserved and human-specific regulation.

Previous cross-species studies showed that similar tissues/organs exhibit stronger gene expression correlations than different tissues/organs within the same species (63, 64). However, we demonstrate here that both the AT transcriptome and 3D genome varied more between species than between ATs of the same species. This highlights species-specific changes in the molecular regulatory network among mammalian ATs. By exploring the characteristics of chromatin architecture at different genomic scales, we revealed that 3D chromatin organizations are more dynamic between species at the level of fine-scale PEIs compared to large-scale TADs and compartments. A previous study found an increasing amount of changes along the genome architectural hierarchy (from compartments to TAD, sub-TAD, and PEI) during cell differentiation (65). Our data suggest that 3D genome organizations are perhaps more extensively reorganized locally, not only across cell types but also across species. Furthermore, our results reveal that HS chromatin structures are associated with DNA sequence variation in humans. Notably, we identified several TFs as potential mediators for HS-PEIs in human ATs. Their binding motifs were less conserved in HS-PEIs, especially in promoter regions. Compared with enhancers, the evolutionary rate of promoters is supposedly slower across mammalian species (66, 67, 68). However, we propose that evolutionary changes in TF binding motifs in promoters may determine the target gene of rapidly evolved enhancers, thereby contributing to the precise regulation of gene expression. These findings highlight the potential role of evolutionarily changed motifs in 3D genome evolution.

In the past, some studies identified multiple regulatory phenotypes that contribute to gene expression divergence between species (69, 70, 71, 72). We found the evolution of chromatin structures is closely linked to the evolution of gene expression in ATs. Our observations suggest that the 3D genome conformation is one of the putative upstream factors involved in the evolution of gene regulation. Enhancers have long been associated with an additive effect on target gene expression (41, 42). However, we observed the formation of HS-PEIs only slightly up-regulates gene expression in humans. This is likely due to the following two reasons. First, we observed that HS-PEIs enhancers exhibit lower enhancer activity compared to Cons-PEIs, which is consistent with previous findings that showed evolved enhancers are functionally weaker than conserved ones across species (73). Second, we found many promoters regulated by multiple Cons-PEIs, which may provide robustness to gene expression across species by working as a buffer against perturbations (74, 75). Hence, the formation of human-specific PEIs is likely a fine-tuning mechanism for the phenotypic evolution of human ATs. Given the low 10-kb resolution of the Hi-C data used in the PEI analysis, it is also possible that we have underestimated the contribution of human-specific PEIs to gene expression differences between species. Future comparative Hi-C studies with higher, sub-kilobase resolutions, will make it possible to resolve the variation of PEI structures at even smaller scales.

Notably, all three classes of HS 3D chromatin structures (*i.e.*, compartment, TAD, and PEI) are enriched for genes associated with energy metabolism, possibly reflecting regulatory differences in metabolic processes between humans and other mammals. Typically, all four genes related to “positive regulation of fatty acid oxidation” process were regulated by HS PEIs. Additionally, genes participating in glucose metabolism, such as *GLIS3* (32) and *ERBB4* (76) were also associated with HS 3D chromatin structures. This is potentially related to adopting a sugar and lipid preference diet in humans, which is accompanied by a high energy demands of the larger brain (77, 78). Furthermore, we observed that a large proportion gene (11 of 17) related to “unsaturated fatty acid metabolic process” are regulated by HS-PEIs in human SAT, this is consistent with the idea that improvements in consumption of dietary long-chain polyunsaturated fatty acids are necessary for promoting encephalization of humans (77, 79). Hence, the identified HS 3D chromatin structures in ATs could provide novel insights into the understanding of the genetic basis underlying human metabolic innovations. In addition, the HS PEIs may also provide a candidate set of *cis*-regulatory elements that may contribute to metabolic variation between humans and other mammals. Compared to HS chromatin organizations, 3D chromatin structures conserved across seven mammalian SATs are more enriched for housekeeping genes, which are essential for the existence of cells. The thermogenesis of ATs is an essential survival mechanism for homeotherms, which allows for an increase in whole-body energy expenditure and is associated with beneficial metabolic effects (3, 9). Our study found that thermogenesis-related genes (typically, *GPR3* (80) and *EBF2* (81)) are preferentially regulated by Cons PEIs rather than HS PEIs. Furthermore, genes such as *FOS* (82) and *VEGFA* (83), along with genetic variants associated with human health, are also more enriched in Cons PEIs relative to HS PEIs. These findings have allowed us to propose that the evolutionarily conserved chromatin organizations are widely important and may be closely link to the health and survival of different organisms. Of note, the epigenetic modifications in chromatin (such as chromatin accessibility, histone modification, and DNA methylation) are also important for transcriptional regulation besides the 3D chromatin structure itself (84, 85, 86). Therefore, more experimental studies and epigenetic data are necessary for functional validation and insights into the regulatory mechanisms of 3D genome organizations during AT evolution.

We found that HS chromatin organizations are less shared among ATs obtained from different anatomical locations than Cons organizations at the TAD and PEI levels. These findings suggest a location-specific evolutionary strategy of ATs at 3D genome level and highlight the importance of defining location-relevant AT chromatin organizations in future studies. We found that VAT-specific HS TADs and HS PEIs are more closely linked to inflammatory and immune processes than those that specifically occurred in SAT, suggesting the immune and inflammatory properties of VATs may have been selected for during evolution. Currently, the immune-related roles of RAD are poorly studied (60). Our results indicate that RAD-specific HS TADs and PEIs are closely related to immune and inflammatory responses, suggesting the immune function of RAD requires further study. We also found that VAT-specific HS PEIs are preferentially associated with metabolic disease, this is consistent with the previous view that VATs are often considered more pernicious than SATs (9). Collectively, our study provides a new perspective for understanding biological differences between SAT and VAT and implies that the root of functional divergence between ATs could be found during AT evolution.

To our knowledge, our work reveals, for the first time, the reorganization of 3D genome architecture during AT evolution. We identified evolutionarily conserved and human-specific chromatin structures in human ATs at different genomic scales. We also demonstrate that the structural dynamics of the 3D genome is closely associated with transcriptome remodeling, underscoring the importance of 3D genome regulation in AT evolution. This 3D regulatory landscape significantly increases current knowledge of the evolution of gene regulatory networks in ATs and provides a new resource for future in-depth evolutionary studies.

## Experimental procedures

### Animals and sample collection

The analyzed SATs (2–3 biological replicates for each mammal) in this study were derived from adult humans (*Homo sapiens*) and six mammals, including mice (*Mus musculus*), rats (*Rattus norvegicus*), rabbits (*Oryctolagus cuniculus*), pigs (*Sus scrofa*), sheep (*Ovis aries*), and dogs (*Canis lupus familiaris*). The visceral adipose tissues (VATs) (*i.e.*, greater omentum [GOM], mesenteric adipose [MAD], and retroperitoneal adipose [RAD]) were derived from humans, pigs, and sheep (3 biological replicates for each VAT of each mammal). Of these, the SATs collected from mice (two pools, 10 mice per pool, Kunming mouse, 32-week-old, females) and VATs (including GOM, MAD, and RAD) collected from sheep (*n* = 3, small-tail Han sheep, 2-year-old, females) were newly generated samples in this study. Animals in this study were handled according to the guidelines of the Administration of Affairs Concerning Experimental Animals established by the Ministry of Science and Technology of China. The Experimental procedures used in the study were approved by the Institutional Animal Care and Use Committee in the College of Animal Science and Technology, Sichuan Agricultural University, Sichuan, China, under permit number DKY-2019102006.

All animals were allowed access to food and water *ad libitum* and were humanely euthanized, when necessary, to ameliorate suffering after 12 h of fasting. All samples were immediately frozen in liquid nitrogen and stored at −80 °C until use in *in situ* Hi-C and rRNA-depleted RNA sequencing (RNA-seq) library preparation.

### *In situ* Hi-C library preparation

In this study, we generated 11 *in situ* Hi-C libraries (Table S1) using the same experimental protocols as the Hi-C dataset that we previously reported (87). Briefly, 1 g of ATs were cross-linked with a final concentration of 4% freshly prepared formaldehyde (Sigma Aldrich) for 30 min at room temperature, followed by quenching with glycine (Amresco) at a final concentration of 0.25 mol l^−1^. After centrifuging the mixture at 1500*g* for 10 min at room temperature, the upper layer containing adipocytes was added to 1 ml lysis buffer (9.1 μl 1 M NaCl, 9.1 μl 1 M Tris-HCl [pH 8.0] [Invitrogen, Carlsbad, CA, USA], 18.2 μl 10% CA-630 [Sigma Aldrich], 50 μl protease inhibitors [Sigma Aldrich], and 913.6 μl nuclease-free water [Ambion]) and homogenized with a Dunce homogenizer. After centrifuging the homogenate at 5000*g*, the cell nuclei were collected. The pellet was washed twice with 500 μl 1 × NEBuffer 2 (NEB), followed by centrifugation at 5000 rpm for 5 min. Next, the pellet was resuspended in 100 μl 1 × NEBuffer 2, and we added SDS (Amresco) to a final concentration of 0.1%. After incubation at 65 °C for 10 min, we added Triton X-100 (Sigma Aldrich) to a final concentration of 1% and incubated it for 15 min at 37 °C. Nuclei were permeabilized, and DNA was digested with 200 units of MboI enzyme (NEB) at 37 °C for 1 h, 65 °C for 20 min, and 25 °C for 5 min. We then added biotin-14-dATP, dCTP, dGTP, dTTP, and Klenow fragment (NEB) and incubated the mixture at 37 °C for 45 min. The DNA fragments were then ligated by T4 DNA ligase (Enzymatics) and incubated at 20 °C for 30 min. After centrifugation at 5000 rpm for 5 min, the pellet was resuspended in 20 μl 10 × T4 DNA ligase buffer, 90 μl nuclease-free water, and 20 μl SDS and 50 μl Proteinase K (20 mg ml^−1^) (Tiangen) was added. After incubation at 55 °C for 30 min to digest proteins, the DNA was extracted and dissolved in 20 μl of 5 M NaCl, and the mixture was incubated sequentially at 65 °C for 90 min and at 25 °C for 5 min. The DNA was then purified by AMPure XP Beads (Beckman Coulter), after which T4 DNA polymerase (Enzymatics) was used to remove nonligated biotinylated DNA for 2 h at 12 °C. The DNA was then sonicated into 300 to 500 bp fragments using a Covaris S220 sonicator. The DNA fragments with biotin were pulled down by M280 beads (Invitrogen). Next, we performed end repair, A-tailing, adapter ligation, post-ligation cleanup, and PCR amplification (8–10 cycles) using the KAPA Hyper Prep Kit (Roche). DNAs between 300 and 800 bp in size were then isolated using AMPure XP Beads, and the libraries were sequenced using Illumina NovaSeq 6000 (paired-end sequencing with 150 bp read length).

### RNA-seq library preparation and gene expression quantification

We generated nine RNA-seq libraries in this study (Table S1). Total RNA was extracted from each sample using the RNeasy Mini Kit (Qiagen, Valencia, CA, USA). Concentration and purity were quantified by Nanodrop, and integrity was evaluated using an Agilent 2000. RNA-seq libraries were then generated using an rRNA depletion method (Globin-Zero Gold rRNA Removal Kit, Illumina) coupled with a NEBNext Ultra Directional RNA Library Prep Kit for Illumina (NEB). The libraries were sequenced by the HiSeq X 10 platform (Illumina) with a paired-end sequencing length of 150 bp.

The sequence reads of each species were aligned against their respective reference genomes. The gene expression levels were estimated as transcripts per million (TPM) using the Kallisto tool (version 0.44.0) (88) with default parameters. The gene annotation file of each species was downloaded from the Ensembl database (release 103). The reference genome assemblies for seven species included: humans (GRCh38.p13), mice (GRCm39), rats (Rnor_6.0), rabbits (OryCun2.0), pigs (Sscrofa11.1), sheep (Oar_rambouillet_v1.0), and dogs (CanFam3.1).

### Hi-C data preprocessing and normalization

Hi-C read pairs were processed using the Juicer pipeline (version 1.5.6) (89) as described in a previous report. Read pairs of seven species were aligned against their respective reference genomes using BWA software (version 0.7.8) (90) with default parameters. Invalid read pairs were filtered out, including PCR duplications, low-quality alignment read pairs (MAPQ < 30), and intrafragment read pairs. To explore the similarity of the chromatin conformation among replicates or different ATs within each species, we generated the raw intra-chromosomal contact matrices using valid read pairs at 100 kb, which were then normalized using the Knight-Ruiz (KR) algorithm (89, 91) and quantile algorithm (92). The similarity between normalized contact matrices was evaluated using HiCRep (93). To perform an interspecies comparison of genome architecture for ATs, we merged biological replicates and down-sampled the Hi-C dataset to similar coverage of valid contacts for each comparison. We generated the normalized intra-chromosomal Hi-C contact maps at various resolutions (10-, 20-, and 100-kb) using the KR algorithm.

### Identification of A | B compartments

A and B compartments were identified at 20-kb resolution by two steps as described in a previous report (26) with minor modifications. First, we generated observed/expected contact matrices at 100-kb resolution by dividing the observed contact frequency by the expected contact frequency (the median observed contact frequency at the same genomic distance). The PC1 values were then calculated using the “prcomp” function in R (version 3.6.1) on the observed/expected contact matrices at 100 kb resolution. Next, A and B compartments were determined using Pearson’s *r* between PC1 values and the gene density of each chromosome. For chromosomes with a positive Pearson’s *r*, the 100 kb bins with positive or negative PC1 vectors were identified as low-resolution A or B compartment. In contrast, for chromosomes with a negative Pearson’s *r*, the 100 kb bins with positive or negative PC1 vectors were identified as low-resolution B or A compartment.

Second, we calculated the A-B index (the likelihood of a sequence interacting with the A or B compartments) at 20 kb resolution. Briefly, for each 20 kb bin, we calculated the median of the observed/expected contact frequency between this 20 kb bin and the other low-resolution A compartment regions or low-resolution B compartment on its chromosome at 20 kb resolution as the A score or B score. The A-B index was then obtained by subtracting the B score from the A score. The higher the A-B index, the more inclined this region is toward the A compartment state.

### Recognition of evolutionary patterns of compartmentalization

Evolutionary patterns of compartmentalization were identified using the Phylo-HMGP method as described in a previous report (28) with minor modifications. This enables the classification of genomic regions into a predefined number of states, which considers both spatial dependencies along the entire genome and temporal dependencies across species in the phylogeny. The human genome was used as the “reference” and divided into 20-kb bins. The homologous genomic regions shared by all seven mammalian species were identified using the UCSC liftOver tool at 20 kb resolution. For example, if one 20-kb bin of the human genome was aligned to all other six species with reciprocal mapping, this bin was identified as homologous regions. According to these homologous regions, we generated an A-B index matrix. To apply Phylo-HMGP to these A-B index data, we first performed K-means clustering to the datasets with an increasing cluster number of K. We computed the Sum of Squared Error (SSE) of each clustering result and observed that the ‘elbow point’ of the SSE curve is around seven, which we considered being the number of evolutionary states. Finally, the A-B index matrix was used to calculate evolutionary patterns implemented in the original public code (https://github.com/ma-compbio/Phylo-HMGP) of the Phylo-HMGP with the state number set to be seven. Other parameters are default.

The evolutionary patterns of compartmentalization across three species (humans, pigs, and sheep) for each of the four ATs (SAT, GOM, MAD, and RAD) were identified similarly.

### TAD calling

We identified TAD boundaries using a previously reported insulation score (IS) method (27) based on the KR normalized intra-chromosomal Hi-C contact maps at 20-kb resolution. The IS reflects the aggregate of contacts passing across each bin, and TAD boundaries were called using the public code (matrix2insulation.pl, https://github.com/dekkerlab/cworld-dekker) with parameters (-v -is 260,000 -ids 200,000 -im mean -nt 0.5 -bmoe 0).

### Calculation of TAD strength

We calculated the TAD strength according to methods described in a previous study (94). We defined the neighbor region of each TAD by extending upstream and downstream of the TAD by its same length and calculated the median of the ratios of intra-TAD contacts (occurred within the TAD region) to inter-TAD contacts (occurred between the TAD region and its neighbor region) across various genomic distances within a TAD as TAD strength. The formula for the TAD strength is below:

$$TAD\;strength=median\left(\frac{intra-TAD\left(d_{i}\right)\;contacts}{inter-TAD\left(d_{i}\right)\;contacts}\right)$$

The intra- and inter-TAD (d_i_) contacts represented the median of intra-TAD contacts and the median of inter-TAD contacts at a certain genomic distance (d_i_), respectively (see the illustration below).

**Figure:**
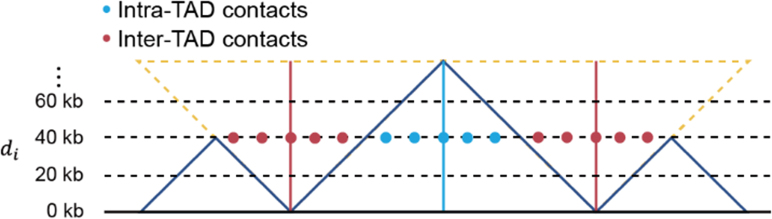

### Identification of species-conserved and species-specific boundaries and TADs

Species-conserved and species-specific TAD boundaries were identified as described in a previous report (16) with minor modifications. We first identified species-conserved and human-specific boundaries by the following pipeline. We converted the TAD boundaries of humans (reference species) to each of the other six mammalian species (query species) using the UCSC liftOver tool. If the human boundary aligned within the flanking 40-kb (2 bins) region of the boundary in another species, the two boundaries were defined as conserved between the two species (see the illustration below). The species-conserved boundary was defined as those conserved across all seven species. If the distance between the human boundary and that of the other six species was greater than or equal to 100 kb (5 bins), the boundary was defined as human-specific. The same pipeline was used to identify species-specific boundaries of non-human species. Finally, a species-conserved TAD is flanked by the species-conserved boundaries on both sides; a species-specific TAD is flanked by the species-specific boundaries at least on one side.

The identification of species-conserved and human-specific TAD boundaries and TADs across three species (humans, pigs and sheep) for the four ATs (SAT, GOM, MAD, and RAD) was performed in a similar manner.

**Figure:**
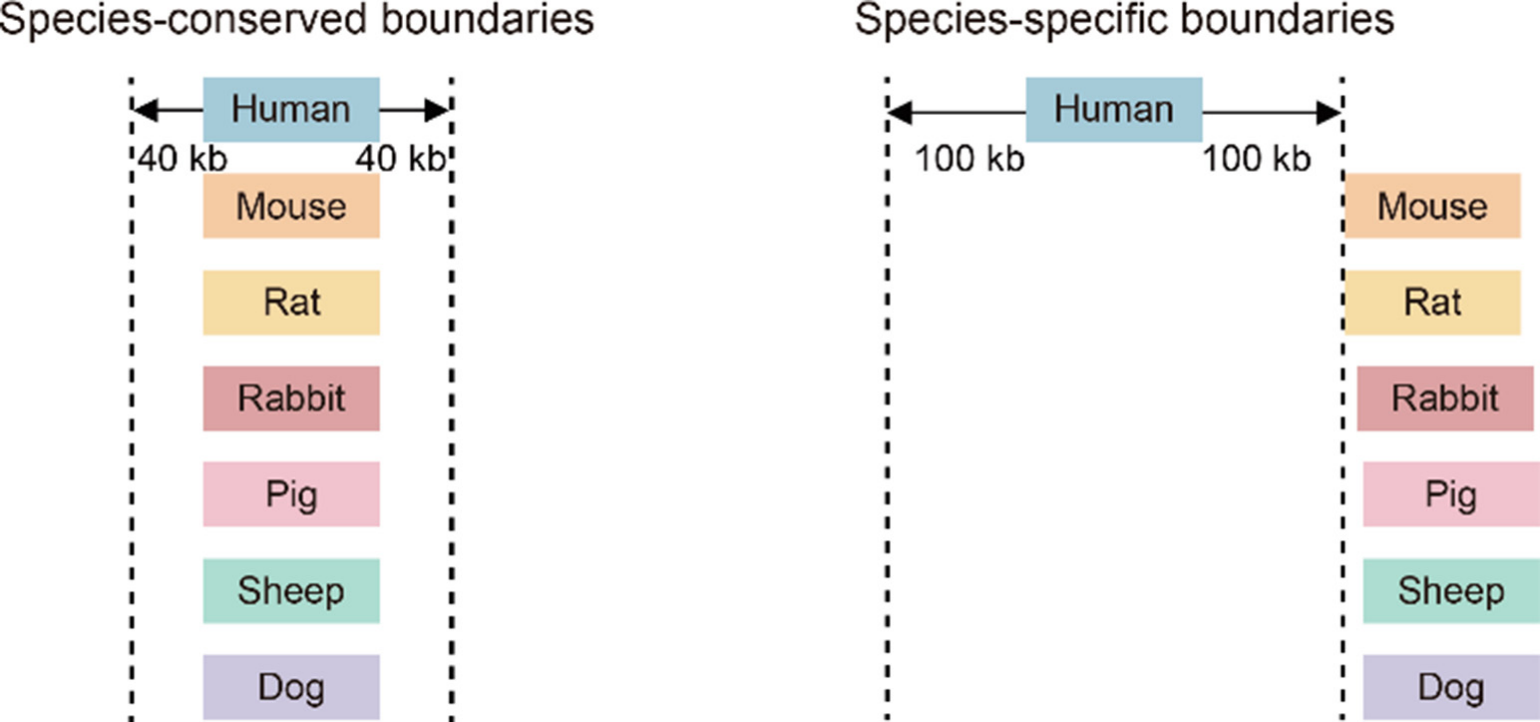

### PEI calling

We detected the overrepresented PEIs using the PSYCHIC algorithm (39), with default parameters as previously described, based on KR-normalized intra-chromosomal contact matrices at 10 kb resolution. We first defined the promoters as upstream 2200 bp and downstream 500 bp of the transcription start site. We then compared the observed contact frequency of promoter-centered interactions (±10 Mb) with its expected contact frequency (defined as the normalized contact frequency according to the domain-specific background model, output by PSYCHIC). The statistical significance score (*p* values and false discovery rate [FDR] values) was calculated, and the output was generated by PSYCHIC. Finally, we filtered the interactions between promoters and applied a cutoff with an FDR value ≤ 0.001 and interaction length ≥ 20 kb to obtain high-confidence PEIs. To assess the active state of enhancers in PEIs, we downloaded the public H3K27ac ChIP-seq peaks (including regular-enhancer [RE] peaks and super-enhancer [SE] peaks) of human SATs. REs and SEs were considered to be involved in PEIs if the 10 kb enhancer bin overlapped more than 50% (or more than 5 kb length) of RE or SE peaks.

To evaluate the enrichment of the identified PEIs, the aggregated peak analysis (APA) scores were calculated by Juicer (version 1.5.6) (89) with a bin size of 10 kb. APA scores greater than one represent enrichment peak.

### Identification of species-conserved and species-specific PEIs

The species-conserved and species-specific PEIs were identified as follows. We first selected the human PEIs associated with single-copy orthologous genes across seven mammals for downstream analyses. Then, the coordinates of these human PEIs (reference species) were converted to each of the other six species genome (query species) using the UCSC liftOver tool. PEIs with both anchors were aligned to two regions ranging from 5 to 20 kb (within the same chromosome) with reciprocal mapping were used for subsequent analysis. Converted promoter coordinates that were less than 20 kb away from its homologous promoter in the other six species genomes were retained. We then performed two kinds of judgments to define each human PEI as one species-conserved or human-specific PEI, including the distance judgment, which confirmed whether the aligned PEI is near any PEIs that regulated its homologous promoter in the query species, and the enrichment judgment, which confirms whether the aligned PEI show signal enrichment in the intra-chromosomal contact matrix of the query species.

The distance judgment was defined by comparing the two-dimensional Euclidean distance between two PEIs with the following thresholds:

$$d<\min\left(r_{1}\times \left|i-j\right|,r_{2}\right)$$

The d represented the two-dimensional Euclidean distance between an aligned PEI and its closest PEI in the corresponding query species and the |i−j| represented the linear distance between two anchors of the aligned PEI in query species. The $r_{1}$ and $r_{2}$ were relative distance coefficient and absolute distance threshold, respectively, and 0.2 and 20 kb were used to identify species-conserved PEIs, while 0.2 and 50 kb were used to identify species-specific PEIs.

The enrichment judgment was defined to check whether the aligned PEIs showed strong signal enrichment in query species. We calculated the enrichment score of all PEIs in the corresponding query species, which was calculated as observed contact frequency minus its expected contact frequency (output by PSYCHIC). We selected 20% quantiles as the threshold. The aligned PEIs whose enrichment score exceeded this threshold were considered signal enriched.

Combining the distance and enrichment judgment, an aligned PEI was defined as a species-conserved or human-specific PEI according to the following: If an aligned PEI was close enough to a PEI in the query species that involved its homologous promoter or showed signal enrichment, it was defined as a species-conserved PEI; if an aligned PEI was far from all PEI associated with its homologous promoter in query species and had no signal enrichment, it was defined as a human-specific PEI. The same pipeline was used to identify species-specific PEIs in other mammals.

The identification of species-conserved and human-specific PEIs across three species (humans, pigs, and sheep) for four ATs (SAT, GOM, MAD, and RAD) was performed with the same procedure.

### Chromatin 3D modeling

The local 3D chromosome conformations were inferred based on the KR normalized intra-chromosomal Hi-C contact maps (at 10 kb resolution) by using an approximation of the multidimensional scaling (MDS) method as implemented in the miniMDS Python package (https://github.com/seqcode/miniMDS) (95) with default parameters. PyMOL (version 2.5.2) was used to simulate the local 3D chromatin conformation of the genome.

### Detection of TF binding motifs

The TF binding motif enrichment analysis for the ATAC-seq peaks within species-conserved and human-specific PEIs was performed using the Analysis of Motif Enrichment tool (96) within the MEME suite (version 5.4.1) (97) with default settings. HOCOMOCO Human (version 11 CORE) database (98) was selected as the source of the motifs. For motif occurrences, FIMO (version 5.1.1) (99) was used with default parameters. The TFs whose binding displayed human-specific or species-conserved PEIs preference were determined by the difference of motif enrichment *p* value (−log_10_) between HS and species-conserved PEIs (|△| > 5).

### Identification and visualization of TF pairs associated with HS PEI formation

The TF pairs potentially involved in human-specific PEI formation were identified using a computational framework described in a previous study (100) with minor modifications. The process involves the following steps (1): TF motifs that occurred at least three times within ATAC-seq peaks of promoter or enhancer were considered, the analytical procedures of motif occurrence are described in “Detection of transcription factor binding motifs” (2); the expression of TF genes require TPM > 1 in human SATs (3); the predicted TF pairs between promoter and enhancer can recover direct PPIs or indirect PPIs (PPI separated by one protein in the PPI network, since intermediate proteins could be involved in PEIs formation) in the STRING database (version 11.5) (55). To identify the significant TF pairs that occur in human-specific PEIs rather than species-conserved PEIs, a Mann-Whitney U-test was used with *p* < 0.05 as the cutoff.

### Identification of expanded gene families

The expanded gene families were identified using computational analysis of gene family evolution (CAFE) as described in a previous report (101) with minor modifications. A specified phylogenetic tree of seven species (including humans, mice, rats, rabbits, pigs, sheep, and dogs) was obtained from the TimeTree database (102). We calculated the gene family size in each species based on the homologous genes across the seven species, which were downloaded from the Ensembl database (release 103). Then, the specified phylogenetic tree and the gene family size in each of the seven species were used as inputs to perform a statistical analysis of the evolution of the size of gene families using the public CAFE software (http://www.bio.indiana.edu/∼hahnlab/Software.html) with default parameters. The expanded gene family for each species was defined using the criteria *p* value < 0.05.

### Gene expression normalization

The gene expressions of single-copy orthologous genes across seven species were normalized using a scaling procedure as described in a previous report (63). The gene expressions of single-copy orthologous genes were first log_2_-transformed. Then, using single-copy orthologous genes whose expression is in the interquartile range, we identified 1000 single-copy orthologous genes with the most-conserved ranks among samples. We calculated the median expression levels of these most-conserved single-copy orthologous genes in each sample and derived scaling factors that adjusted these medians to a common value. Finally, the expression level of all single-copy orthologous genes was normalized among samples using these scaling factors.

### Functional enrichment analysis

Functional enrichment of specific gene sets was performed using Metascape (http://metascape.org) (103) with default settings. Gene Ontology (GO)-biological processes (GO-BP) and Kyoto Encyclopedia of Genes and Genomes (KEGG) pathways were selected as ontology sources. The most statistically significant terms in each cluster are shown.

### Statistical analyses

For all the bar graphs, data are represented as mean ± SD. All statistical analyses were performed in R (version 3.6.1), and statistical details can be found in the figure legend.

## Data availability

The RNA-seq and *in situ* Hi-C dataset generated in this study are available at Gene Expression Omnibus (GEO) database (GSE199968, https://www.ncbi.nlm.nih.gov/geo/query/acc.cgi?acc=GSE199968). Sources of public datasets analyzed during the current study are listed in Table S1.

## Supporting information

This article contains supporting information: Tables S1–S16, Figures S1–S12.

## Conflict of interest

The authors declare that they have no conflicts of interest with the contents of this article.

## References

[1] Wajchenberg B.L. (2000). Subcutaneous and visceral adipose tissue: their relation to the metabolic syndrome. Endocr. Rev..

[2] Lee M.J., Wu Y., Fried S.K. (2013). Adipose tissue heterogeneity: implication of depot differences in adipose tissue for obesity complications. Mol. Aspects. Med..

[3] Sakers A., De Siqueira M.K., Seale P., Villanueva C.J. (2022). Adipose-tissue plasticity in health and disease. Cell.

[4] Hajer G.R., van Haeften T.W., Visseren F.L. (2008). Adipose tissue dysfunction in obesity, diabetes, and vascular diseases. Eur. Heart J..

[5] Bluher M. (2009). Adipose tissue dysfunction in obesity. Exp. Clin. Endocrinol. Diabetes.

[6] Donohoe C.L., Lysaght J., O'Sullivan J., Reynolds J.V. (2017). Emerging concepts linking obesity with the hallmarks of cancer. Trends. Endocrinol. Metab..

[7] Dietz W., Santos-Burgoa C. (2020). Obesity and its implications for COVID-19 mortality. Obesity.

[8] Lafontan M., Girard J. (2008). Impact of visceral adipose tissue on liver metabolism. Part I: Heterogeneity of adipose tissue and functional properties of visceral adipose tissue. Diabetes Metab..

[9] Morigny P., Boucher J., Arner P., Langin D. (2021). Lipid and glucose metabolism in white adipocytes: pathways, dysfunction and therapeutics. Nat. Rev. Endocrinol..

[10] Walker G.E., Marzullo P., Ricotti R., Bona G., Prodam F. (2014). The pathophysiology of abdominal adipose tissue depots in health and disease. Horm. Mol. Biol. Clin. Investig..

[11] Karastergiou K., Fried S.K. (2017). Cellular mechanisms driving sex differences in adipose tissue biology and body shape in humans and mouse models. Adv. Exp. Med. Biol..

[12] Shimizu I., Yoshida Y., Minamino T. (2015). Maintenance of subcutaneous fat homeostasis improves systemic metabolic dysfunction in obesity. Diabetes.

[13] Liu Y., Ge X., Dou X., Guo L., Liu Y., Zhou S.R. (2015). Protein inhibitor of activated STAT 1 (PIAS1) protects against obesity-induced insulin resistance by inhibiting inflammation cascade in adipose tissue. Diabetes.

[14] Sjostedt E., Zhong W., Fagerberg L., Karlsson M., Mitsios N., Adori C. (2020). An atlas of the protein-coding genes in the human, pig, and mouse brain. Science.

[15] Chen H., Li C., Zhou Z., Liang H. (2018). Fast-evolving human-specific neural enhancers are associated with aging-related diseases. Cell. Syst..

[16] Luo X., Liu Y., Dang D., Hu T., Hou Y., Meng X. (2021). 3D Genome of macaque fetal brain reveals evolutionary innovations during primate corticogenesis. Cell.

[17] Ma S., Yim S.H., Lee S.G., Kim E.B., Lee S.R., Chang K.T. (2015). Organization of the mammalian metabolome according to organ function, lineage specialization, and longevity. Cell Metab..

[18] Zwick R.K., Guerrero-Juarez C.F., Horsley V., Plikus M.V. (2018). Anatomical, physiological, and functional diversity of adipose tissue. Cell Metab..

[19] Zuriaga M.A., Fuster J.J., Gokce N., Walsh K. (2017). Humans and mice display opposing patterns of "browning" gene expression in visceral and subcutaneous white adipose tissue depots. Front. Cardiovasc. Med..

[20] Sun W., Dong H., Balaz M., Slyper M., Drokhlyansky E., Colleluori G. (2020). snRNA-seq reveals a subpopulation of adipocytes that regulates thermogenesis. Nature.

[21] Swain-Lenz D., Berrio A., Safi A., Crawford G.E., Wray G.A. (2019). Comparative analyses of chromatin landscape in white adipose tissue suggest humans may have less beigeing potential than other primates. Genome Biol. Evol..

[22] Won H., Huang J., Opland C.K., Hartl C.L., Geschwind D.H. (2019). Human evolved regulatory elements modulate genes involved in cortical expansion and neurodevelopmental disease susceptibility. Nat. Commun..

[23] Won H., de la Torre-Ubieta L., Stein J.L., Parikshak N.N., Huang J., Opland C.K. (2016). Chromosome conformation elucidates regulatory relationships in developing human brain. Nature.

[24] Jin L., Tang Q., Hu S., Chen Z., Zhou X., Zeng B. (2021). A pig BodyMap transcriptome reveals diverse tissue physiologies and evolutionary dynamics of transcription. Nat. Commun..

[25] Jin L. (2022).

[26] Rowley M.J., Nichols M.H., Lyu X., Ando-Kuri M., Rivera I.S.M., Hermetz K. (2017). Evolutionarily conserved principles predict 3D chromatin organization. Mol. Cell.

[27] Crane E., Bian Q., McCord R.P., Lajoie B.R., Wheeler B.S., Ralston E.J. (2015). Condensin-driven remodelling of X chromosome topology during dosage compensation. Nature.

[28] Yang Y., Gu Q., Zhang Y., Sasaki T., Crivello J., O'Neill R.J. (2018). Continuous-trait probabilistic model for comparing multi-species functional genomic data. Cell Syst..

[29] Eisenberg E., Levanon E.Y. (2013). Human housekeeping genes, revisited. Trends. Genet..

[30] Park Y.S., Uddin M.J., Piao L., Hwang I., Lee J.H., Ha H. (2016). Novel role of endogenous catalase in macrophage polarization in adipose tissue. Med. Inflamm..

[31] Akl M.G., Fawzy E., Deif M., Farouk A., Elshorbagy A.K. (2017). Perturbed adipose tissue hydrogen peroxide metabolism in centrally obese men: association with insulin resistance. PLoS One.

[32] Hu C., Zhang R., Wang C., Wang J., Ma X., Hou X. (2010). Variants from GIPR, TCF7L2, DGKB, MADD, CRY2, GLIS3, PROX1, SLC30A8 and IGF1 are associated with glucose metabolism in the Chinese. PLoS One.

[33] Wen X., Yang Y. (2017). Emerging roles of GLIS3 in neonatal diabetes, type 1 and type 2 diabetes. J. Mol. Endocrinol..

[34] Senee V., Chelala C., Duchatelet S., Feng D., Blanc H., Cossec J.C. (2006). Mutations in GLIS3 are responsible for a rare syndrome with neonatal diabetes mellitus and congenital hypothyroidism. Nat. Genet..

[35] Dixon J.R., Selvaraj S., Yue F., Kim A., Li Y., Shen Y. (2012). Topological domains in mammalian genomes identified by analysis of chromatin interactions. Nature.

[36] Rudan M.V., Barrington C., Henderson S., Ernst C., Odom D.T., Tanay A. (2015). Comparative Hi-C reveals that CTCF underlies evolution of chromosomal domain architecture. Cell Rep..

[37] Lazar N.H., Nevonen K.A., O'Connell B., McCann C., O'Neill R.J., Green R.E. (2018). Epigenetic maintenance of topological domains in the highly rearranged gibbon genome. Genome Res..

[38] Schoenfelder S., Fraser P. (2019). Long-range enhancer-promoter contacts in gene expression control. Nat. Rev. Genet..

[39] Ron G., Globerson Y., Moran D., Kaplan T. (2017). Promoter-enhancer interactions identified from Hi-C data using probabilistic models and hierarchical topological domains. Nat. Commun..

[40] Schoenfelder S., Furlan-Magaril M., Mifsud B., Tavares-Cadete F., Sugar R., Javierre B.M. (2015). The pluripotent regulatory circuitry connecting promoters to their long-range interacting elements. Genome Res..

[41] Whalen S., Truty R.M., Pollard K.S. (2016). Enhancer-promoter interactions are encoded by complex genomic signatures on looping chromatin. Nat. Genet..

[42] Cao Q., Anyansi C., Hu X., Xu L., Xiong L., Tang W. (2017). Reconstruction of enhancer-target networks in 935 samples of human primary cells, tissues and cell lines. Nat. Genet..

[43] Zhang L.L., Liu D.Y., Ma L.Q., Luo Z.D., Cao T.B., Zhong J. (2007). Activation of transient receptor potential vanilloid type-1 channel prevents adipogenesis and obesity. Circ. Res..

[44] Siersbaek R., Madsen J.G.S., Javierre B.M., Nielsen R., Bagge E.K., Cairns J. (2017). Dynamic rewiring of promoter-anchored chromatin loops during adipocyte differentiation. Mol. Cell.

[45] Hao R.H., Guo Y., Wang C., Chen F., Di C.X., Dong S.S. (2022). Lineage-specific rearrangement of chromatin loops and epigenomic features during adipocytes and osteoblasts commitment. Cell Death Differ..

[46] Watanabe K., Stringer S., Frei O., Umicevic Mirkov M., de Leeuw C., Polderman T.J.C. (2019). A global overview of pleiotropy and genetic architecture in complex traits. Nat. Genet..

[47] Zabidi M.A., Stark A. (2016). Regulatory enhancer-core-promoter communication *via* transcription factors and cofactors. Trends. Genet..

[48] Wu Z., Wang S. (2013). Role of kruppel-like transcription factors in adipogenesis. Dev. Biol..

[49] Gluscevic M., Paradise C.R., Dudakovic A., Karperien M., Dietz A.B., van Wijnen A.J. (2020). Functional expression of ZNF467 and PCBP2 supports adipogenic lineage commitment in adipose-derived mesenchymal stem cells. Gene.

[50] Kumari M., Wang X., Lantier L., Lyubetskaya A., Eguchi J., Kang S. (2016). IRF3 promotes adipose inflammation and insulin resistance and represses browning. J. Clin. Invest..

[51] Hu R.D., Zhang W., Li L., Zuo Z.Q., Ma M., Ma J.F. (2021). Chromatin accessibility analysis identifies the transcription factor ETV5 as a suppressor of adipose tissue macrophage activation in obesity. Cell. Death Dis..

[52] Boyle K.B., Hadaschik D., Virtue S., Cawthorn W.P., Ridley S.H., O'Rahilly S. (2009). The transcription factors Egr1 and Egr2 have opposing influences on adipocyte differentiation. Cell. Death Dis..

[53] Birsoy K., Berry R., Wang T., Ceyhan O., Tavazoie S., Friedman J.M. (2011). Analysis of gene networks in white adipose tissue development reveals a role for ETS2 in adipogenesis. Development.

[54] Miyashita H., Kanemura M., Yamazaki T., Abe M., Sato Y. (2004). Vascular endothelial zinc finger 1 is involved in the regulation of angiogenesis: possible contribution of stathmin/OP18 as a downstream target gene. Arterioscler. Thromb. Vasc. Biol..

[55] Szklarczyk D., Gable A.L., Lyon D., Junge A., Wyder S., Huerta-Cepas J. (2019). STRING v11: Protein-protein association networks with increased coverage, supporting functional discovery in genome-wide experimental datasets. Nucl. Acids Res..

[56] Spielmann M., Lupianez D.G., Mundlos S. (2018). Structural variation in the 3D genome. Nat. Rev. Genet..

[57] Doan R.N., Bae B.I., Cubelos B., Chang C., Hossain A.A., Al-Saad S. (2016). Mutations in human accelerated regions disrupt cognition and social behavior. Cell.

[58] Capra J.A., Erwin G.D., McKinsey G., Rubenstein J.L., Pollard K.S. (2013). Many human accelerated regions are developmental enhancers. Philos. Trans. R. Soc. Lond. B. Biol. Sci..

[59] Soutar A.K., Naoumova R.P. (2007). Mechanisms of disease: genetic causes of familial hypercholesterolemia. Nat. Clin. Pract. Cardiovasc. Med..

[60] West-Eberhard M.J. (2019). Nutrition, the visceral immune system, and the evolutionary origins of pathogenic obesity. Proc. Natl. Acad. Sci. U. S. A..

[61] Pond C.M., Symonds M.E. (2017). *Adipose Tissue Biology*.

[62] Pond C.M. (1992). An evolutionary and functional view of mammalian adipose tissue. Proc. Nutr. Soc..

[63] Brawand D., Soumillon M., Necsulea A., Julien P., Csardi G., Harrigan P. (2011). The evolution of gene expression levels in mammalian organs. Nature.

[64] Sudmant P.H., Alexis M.S., Burge C.B. (2015). Meta-analysis of RNA-seq expression data across species, tissues and studies. Genome Biol..

[65] Chen C., Yu W., Tober J., Gao P., He B., Lee K. (2019). Spatial genome re-organization between fetal and adult hematopoietic stem cells. Cell Rep..

[66] Villar D., Berthelot C., Aldridge S., Rayner T.F., Lukk M., Pignatelli M. (2015). Enhancer evolution across 20 mammalian species. Cell.

[67] Vierstra J., Rynes E., Sandstrom R.S., Zhang M., Canfield T.K., Hansen R.S. (2014). Mouse regulatory DNA landscapes reveal global principles of cis-regulatory evolution. Science.

[68] Young R.S., Hayashizaki Y., Andersson R., Sandelin A., Kawaji H., Itoh M. (2015). The frequent evolutionary birth and death of functional promoters in mouse and human. Genome Res..

[69] Loisel D.A., Rockman M.V., Wray G.A., Altmann J., Alberts S.C. (2006). Ancient polymorphism and functional variation in the primate MHC-DQA1 5′ cis-regulatory region. Proc. Natl. Acad. Sci. U. S. A..

[70] Pai A.A., Bell J.T., Marioni J.C., Pritchard J.K., Gilad Y. (2011). A genome-wide study of DNA methylation patterns and gene expression levels in multiple human and chimpanzee tissues. PLoS. Genet..

[71] Zhou X., Cain C.E., Myrthil M., Lewellen N., Michelini K., Davenport E.R. (2014). Epigenetic modifications are associated with inter-species gene expression variation in primates. Genome Biol..

[72] Cain C.E., Blekhman R., Marioni J.C., Gilad Y. (2011). Gene expression differences among primates are associated with changes in a histone epigenetic modification. Genetics.

[73] Berthelot C., Villar D., Horvath J.E., Odom D.T., Flicek P. (2018). Complexity and conservation of regulatory landscapes underlie evolutionary resilience of mammalian gene expression. Nat. Ecol. Evol..

[74] Frankel N., Davis G.K., Vargas D., Wang S., Payre F., Stern D.L. (2010). Phenotypic robustness conferred by apparently redundant transcriptional enhancers. Nature.

[75] Osterwalder M., Barozzi I., Tissieres V., Fukuda-Yuzawa Y., Mannion B.J., Afzal S.Y. (2018). Enhancer redundancy provides phenotypic robustness in mammalian development. Nature.

[76] Zeng F., Wang Y., Kloepfer L.A., Wang S., Harris R.C. (2018). ErbB4 deletion predisposes to development of metabolic syndrome in mice. Am. J. Physiol. Endocrinol. Metab..

[77] Burini R.C., Leonard W.R. (2018). The evolutionary roles of nutrition selection and dietary quality in the human brain size and encephalization. Nutrire.

[78] Leonard W.R., Snodgrass J.J., Robertson M.L., Montmayeur J.P., le Coutre J. (2010). Fat Detection: Taste, Texture, and Post Ingestive Effects.

[79] Leonard W.R., Snodgrass J.J., Robertson M.L. (2007). Effects of brain evolution on human nutrition and metabolism. Annu. Rev. Nutr..

[80] Sveidahl Johansen O., Ma T., Hansen J.B., Markussen L.K., Schreiber R., Reverte-Salisa L. (2021). Lipolysis drives expression of the constitutively active receptor GPR3 to induce adipose thermogenesis. Cell.

[81] Stine R.R., Shapira S.N., Lim H.W., Ishibashi J., Harms M., Won K.J. (2016). EBF2 promotes the recruitment of beige adipocytes in white adipose tissue. Mol. Metab..

[82] Knebel B., Kotzka J., Lehr S., Hartwig S., Avci H., Jacob S. (2013). A mutation in the c-fos gene associated with congenital generalized lipodystrophy. Orphanet J. Rare Dis..

[83] Benjamin L.E. (2001). Glucose, VEGF-A, and diabetic complications. Am. J. Pathol..

[84] Thurman R.E., Rynes E., Humbert R., Vierstra J., Maurano M.T., Haugen E. (2012). The accessible chromatin landscape of the human genome. Nature.

[85] Stricker S.H., Koferle A., Beck S. (2017). From profiles to function in epigenomics. Nat. Rev. Genet..

[86] Allis C.D., Jenuwein T. (2016). The molecular hallmarks of epigenetic control. Nat. Rev. Genet..

[87] Zhang J., Liu P., He M., Wang Y., Kui H., Jin L. (2022). Reorganization of 3D genome architecture across wild boar and Bama pig adipose tissues. J. Anim. Sci. Biotechnol..

[88] Bray N.L., Pimentel H., Melsted P., Pachter L. (2016). Erratum: near-optimal probabilistic RNA-seq quantification. Nat. Biotechnol..

[89] Durand N.C., Shamim M.S., Machol I., Rao S.S., Huntley M.H., Lander E.S. (2016). Juicer provides a one-click system for analyzing loop-resolution Hi-C experiments. Cell Syst..

[90] Li H., Durbin R. (2010). Fast and accurate long-read alignment with Burrows-Wheeler transform. Bioinformatics.

[91] Knight P.A., Ruiz D. (2013). A fast algorithm for matrix balancing. IMA J. Numer. Anal..

[92] Fletez-Brant K., Qiu Y., Gorkin D.U., Hu M., Hansen K.D. (2021). Removing unwanted variation between samples in Hi-C experiments. bioRxiv.

[93] Yang T., Zhang F., Yardimci G.G., Song F., Hardison R.C., Noble W.S. (2017). HiCRep: assessing the reproducibility of Hi-C data using a stratum-adjusted correlation coefficient. Genome Res..

[94] Li D., Ning C., Zhang J., Wang Y., Tang Q., Kui H. (2022). Dynamic transcriptome and chromatin architecture in granulosa cells during chicken folliculogenesis. Nat. Commun..

[95] Rieber L., Mahony S. (2017). miniMDS: 3D structural inference from high-resolution Hi-C data. Bioinform..

[96] McLeay R.C., Bailey T.L. (2010). Motif enrichment analysis: a unified framework and an evaluation on ChIP data. BMC Bioinform..

[97] Bailey T.L., Johnson J., Grant C.E., Noble W.S. (2015). The MEME suite. Nucl. Acids Res..

[98] Kulakovskiy I.V., Vorontsov I.E., Yevshin I.S., Sharipov R.N., Fedorova A.D., Rumynskiy E.I. (2018). HOCOMOCO: towards a complete collection of transcription factor binding models for human and mouse *via* large-scale ChIP-seq analysis. Nucl. Acids Res..

[99] Grant C.E., Bailey T.L., Noble W.S. (2011). Fimo: scanning for occurrences of a given motif. Bioinformatics.

[100] Liu L., Zhang L.R., Dao F.Y., Yang Y.C., Lin H. (2021). A computational framework for identifying the transcription factors involved in enhancer-promoter loop formation. Mol. Ther. Nucl. Acids.

[101] De Bie T., Cristianini N., Demuth J.P., Hahn M.W. (2006). Cafe: a computational tool for the study of gene family evolution. Bioinformatics.

[102] Kumar S., Stecher G., Suleski M., Hedges S.B. (2017). TimeTree: a resource for timelines, timetrees, and divergence times. Mol. Biol. Evol..

[103] Zhou Y., Zhou B., Pache L., Chang M., Khodabakhshi A.H., Tanaseichuk O. (2019). Metascape provides a biologist-oriented resource for the analysis of systems-level datasets. Nat. Commun..

