# Supplementary material for: Comparative three-dimensional genome architectures of adipose tissues provide insight into human-specific regulation of metabolic homeostasis
Source: J Biol Chem. 2023 Apr 27;299(6):104757. doi: 10.1016/j.jbc.2023.104757 (PMC10245122; doi:10.1016/j.jbc.2023.104757)
Supplement: Supplementary figures [file mmc17.pdf]

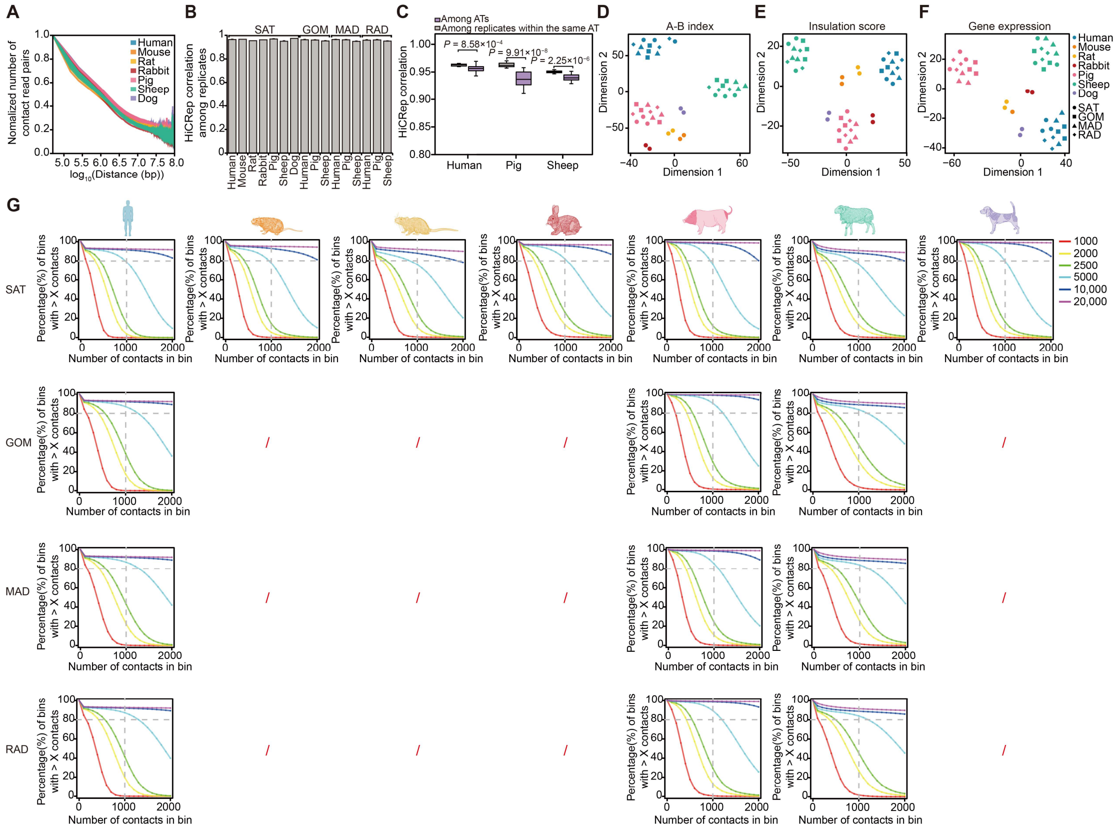

Figure. S1 Basic characterization of Hi-C data from different species.

(A) Log-log contact frequency as a function of genomic distance for each Hi-C library. **(B)** Pairwise correlation of intra-chromosomal Hi-C contacts between biological replicates was calculated using HiCRep at 100 kb resolution. Data are represented as mean ± SD. **(C)** Estimated interrelationship of intra-chromosomal Hi-C matrices between different ATs within human, pig, and sheep species, respectively. **(D-F)** t-SNE visualization of samples based on the A-B index **(D)**, insulation score **(E),** and expression level **(F)** of 9472 single-copy orthologous genes between seven mammals. **(G)** Evaluation of Hi-C resolution. Hi-C map resolutions at various bin sizes (including 1, 2, 2.5, 5, 10, and 20 kb). The maximum resolution was defined as the smallest bin size, where 80% of loci had at least 1000 intra-chromosomal contact reads.

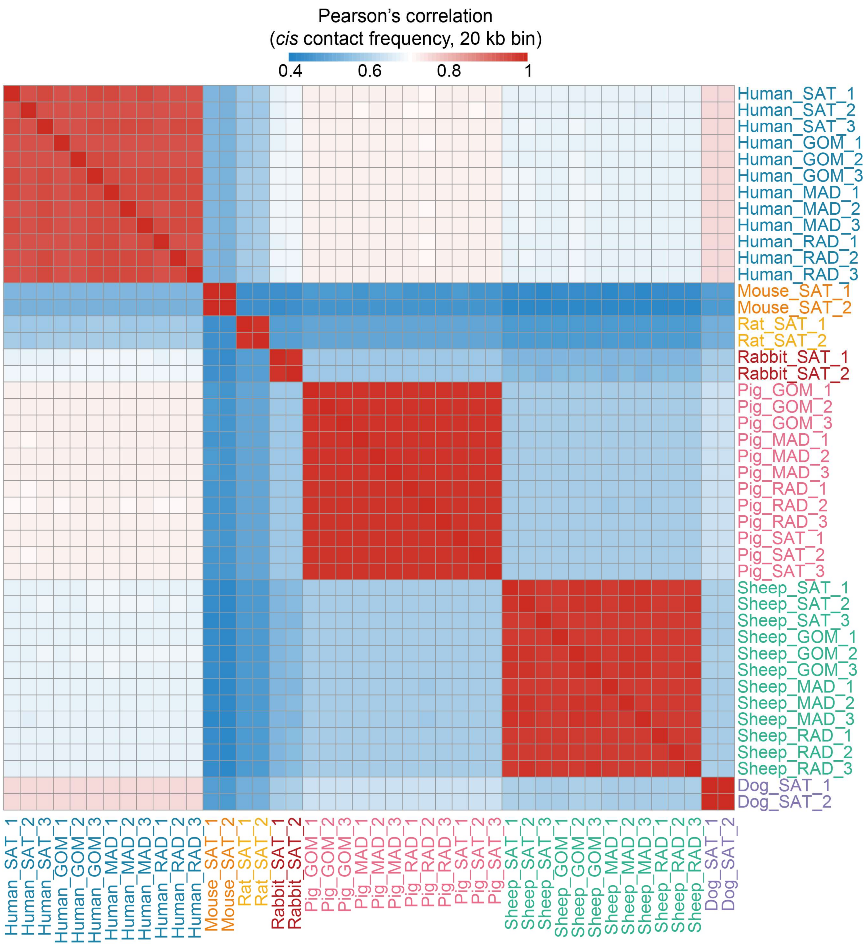

Figure. S2 Overall similarity among all 44 Hi-C datasets. Heat map showing the pairwise Pearson correlation coefficients among all 44 Hi-C datasets, based on the contact frequency of homologous *cis* Hi-C interactions among seven species at 20-kb resolution.

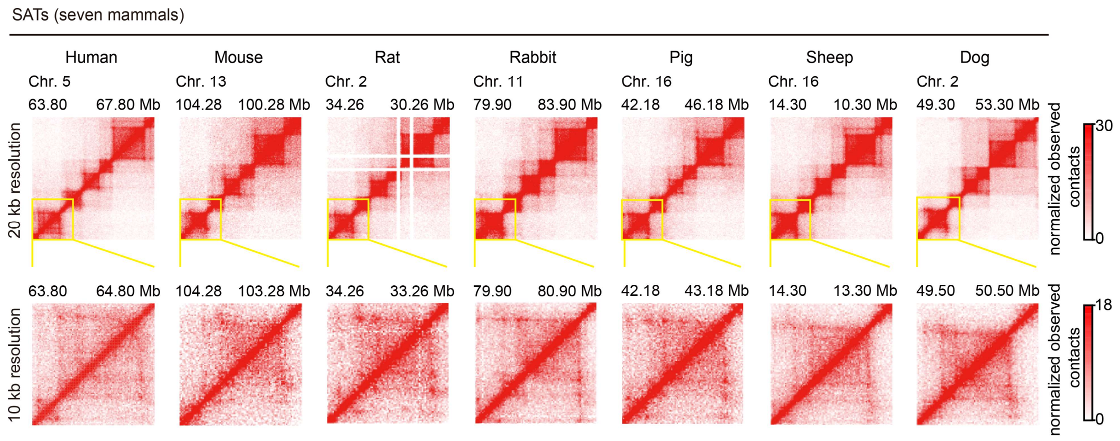

Figure. S3 Illustration of Hi-C contact maps across seven mammalian SATs at 20-kb and 10-kb resolution, respectively. Hi-C maps were normalized using the Knight-Ruiz (KR) algorithm.

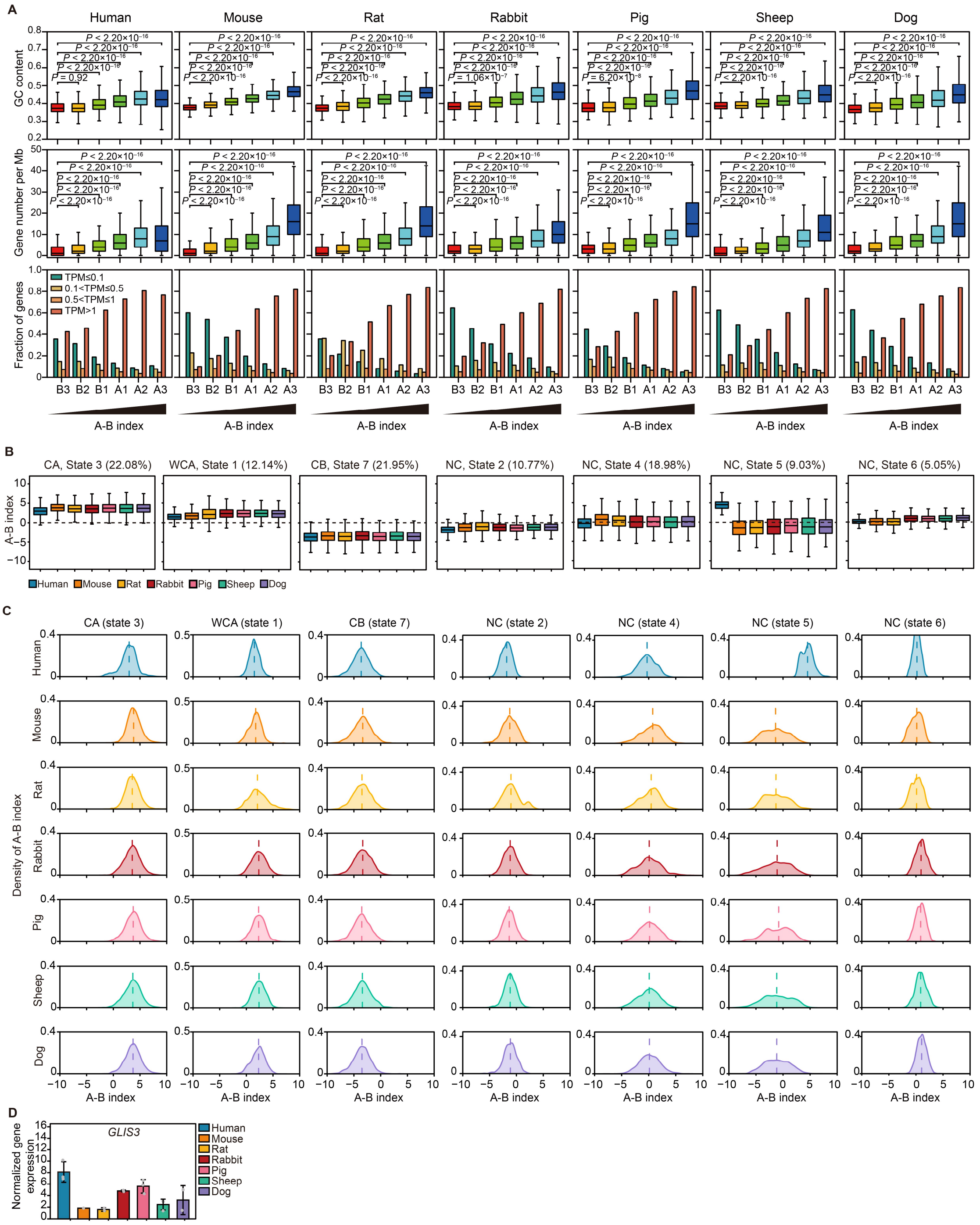

Figure. S4 Characterization of A/B compartment among mammalian SATs.

(A) Distribution of GC content (top panel), gene density (middle panel), and gene expression level (bottom panel) in each mammalian SAT, separated by A-B index categories. A and B compartments were divided into three categories of the same size. The box denotes the 25th to 75th percentile, the horizontal bar is median, whiskers extend to 1.5*IQR beyond the box, and outliers are omitted. *P* values were calculated by Wilcoxon rank-sum test. **(B)** Different patterns of the A-B index across seven mammalian SATs were predicted by Phylo-HMGP for all seven states. Box plots of the

A-B index distributions in seven mammals in each predicted state are shown. It is noted that four compartment states have been shown in Fig. 2A and are shown again here for completeness. **(C)** Density plot of the A-B index of each compartment state in each mammal species. The colored dotted line represents the median A-B index. **(D)** Normalized expression level of the *GLIS3* gene among seven mammalian SATs.

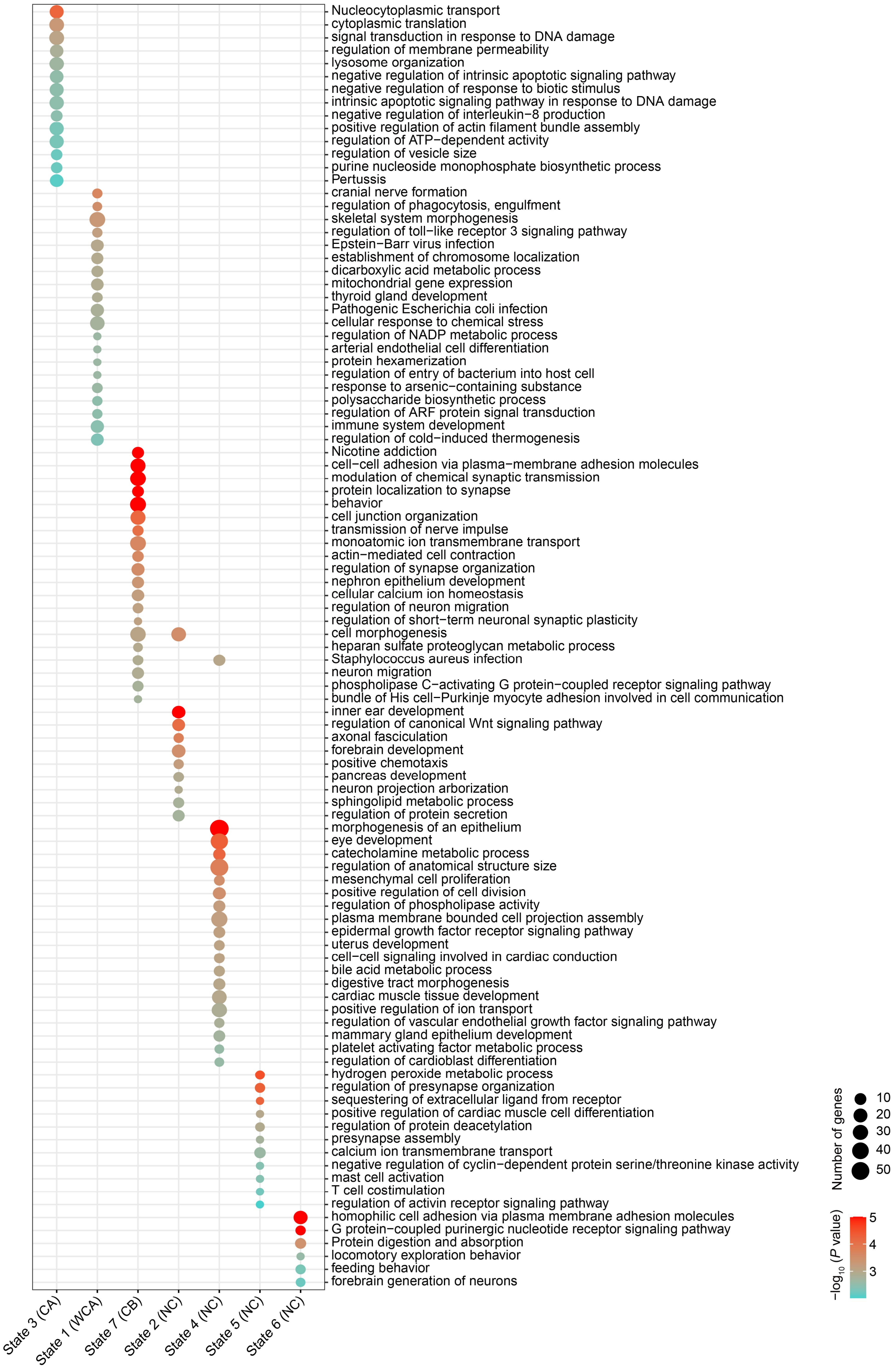

Figure. S5 Functional enrichment analysis of the genes embedded in each compartment state.

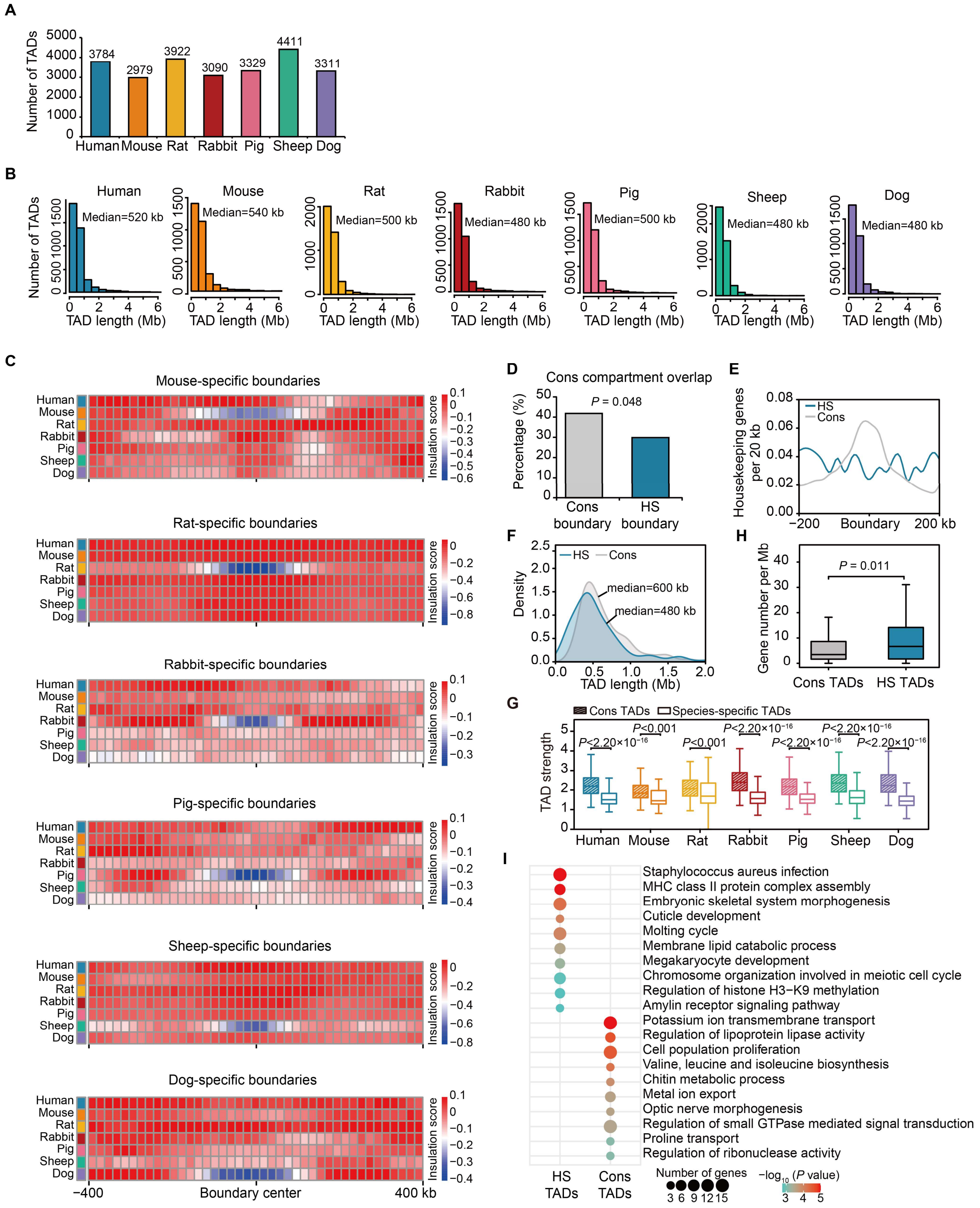

Figure. S6 Characterization of TADs among mammalian SATs.

(A) Number of TADs identified in each mammalian SAT. **(B)** Distribution of TAD length in each mammalian SAT. **(C)** Average insulation score profiles in 400-kb regions centered on species-specific TAD boundaries at 20 kb resolution. **(D)** The percentage of HS and Cons boundaries located within Cons compartments in human SAT. **(E)** Enrichment of housekeeping genes around HS and Cons boundaries in human SAT. **(F)** Distribution of HS and Cons TADs length in human SAT. **(G)** The TAD strength of Cons and respective species-specific TADs for each species. **(H)** Comparison of gene

density between HS and Cons TADs. **(I)** Functional enrichment analysis of genes within HS or Cons TADs.

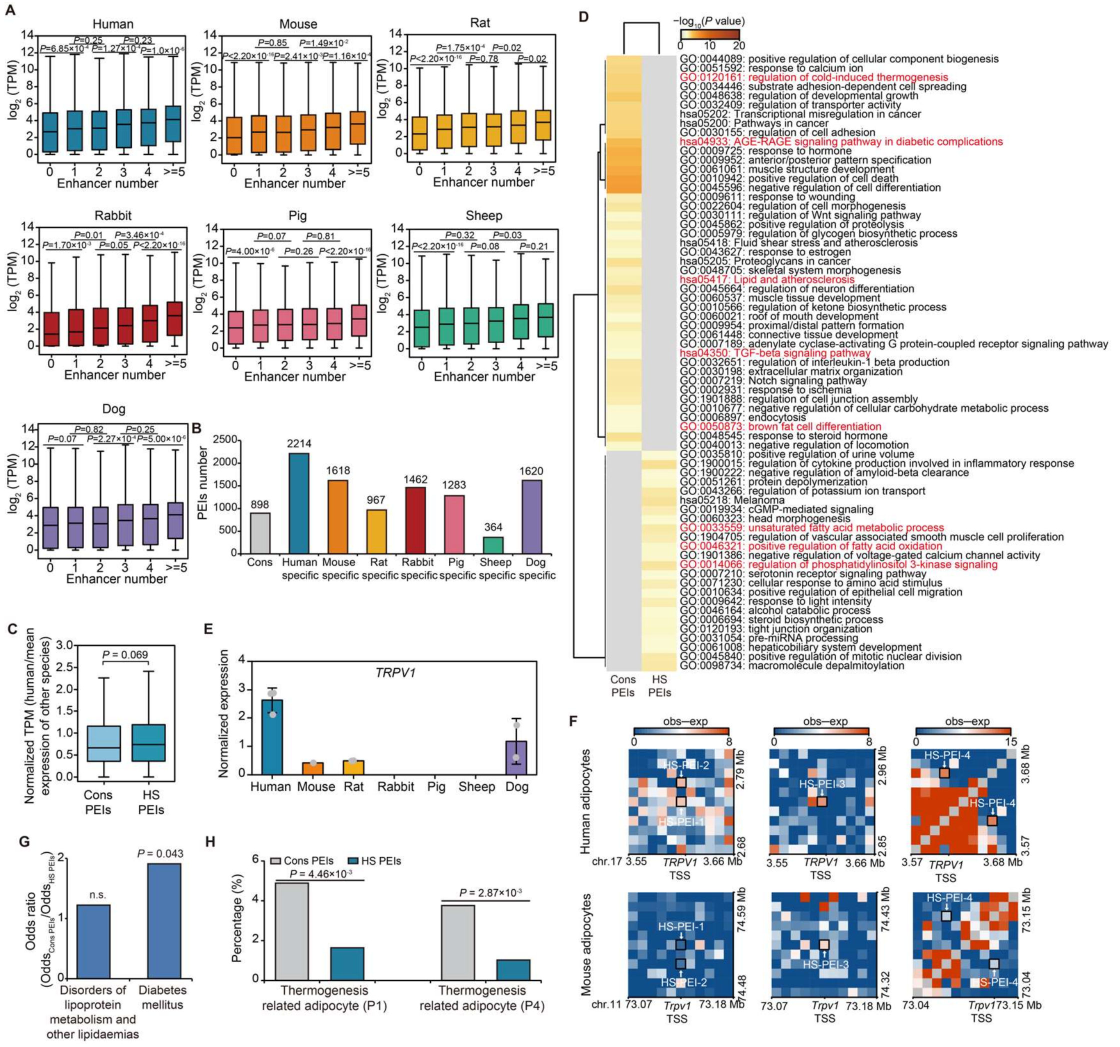

Figure. S7 Characterization of PEIs among mammalian SATs.

(A) Expression level of genes with different interacting enhancer numbers in each mammalian SAT. *P* values were calculated by Wilcoxon rank-sum test. **(B)** The number of Cons and species-specific PEIs. **(C)** Expression change of genes associated with Cons PEIs (n = 378) and HS PEIs (n = 1463) between SATs in humans and other mammals. *P* values were calculated by Student's *t*-test. **(D)** Functional enrichment of genes that are involved in Cons and HS PEIs. **(E)** The normalized expression level of the *TRPV1* gene among seven mammalian SATs. **(F)** Local contact maps around 4 HS PEIs (indicated by black squares) associated with the *TRPV1* gene in humans (upper panel) and mice (lower panel) adipocytes at 10-kb resolution. TSS: transcription start site. **(G)** Odds ratio showing the relationship between enhancer type and SNP enrichment. Odds ratio > 1 indicates that enhancers associated with Cons PEIs tend to enriched SNPs associated with specific traits more than HS PEIs. Fisher's exact test was used to determine significance. n.s., $P \geq 0.05$. **(H)** The percentages of genes regulated by Cons and HS PEIs overlapped with highly-expressed gene sets of two thermogenesis-

related adipocyte subpopulations from the mice adipose tissue, which were named P1 and P4 and exhibited continuously increasing or decreasing compositions in ATs as temperature decreased, respectively.

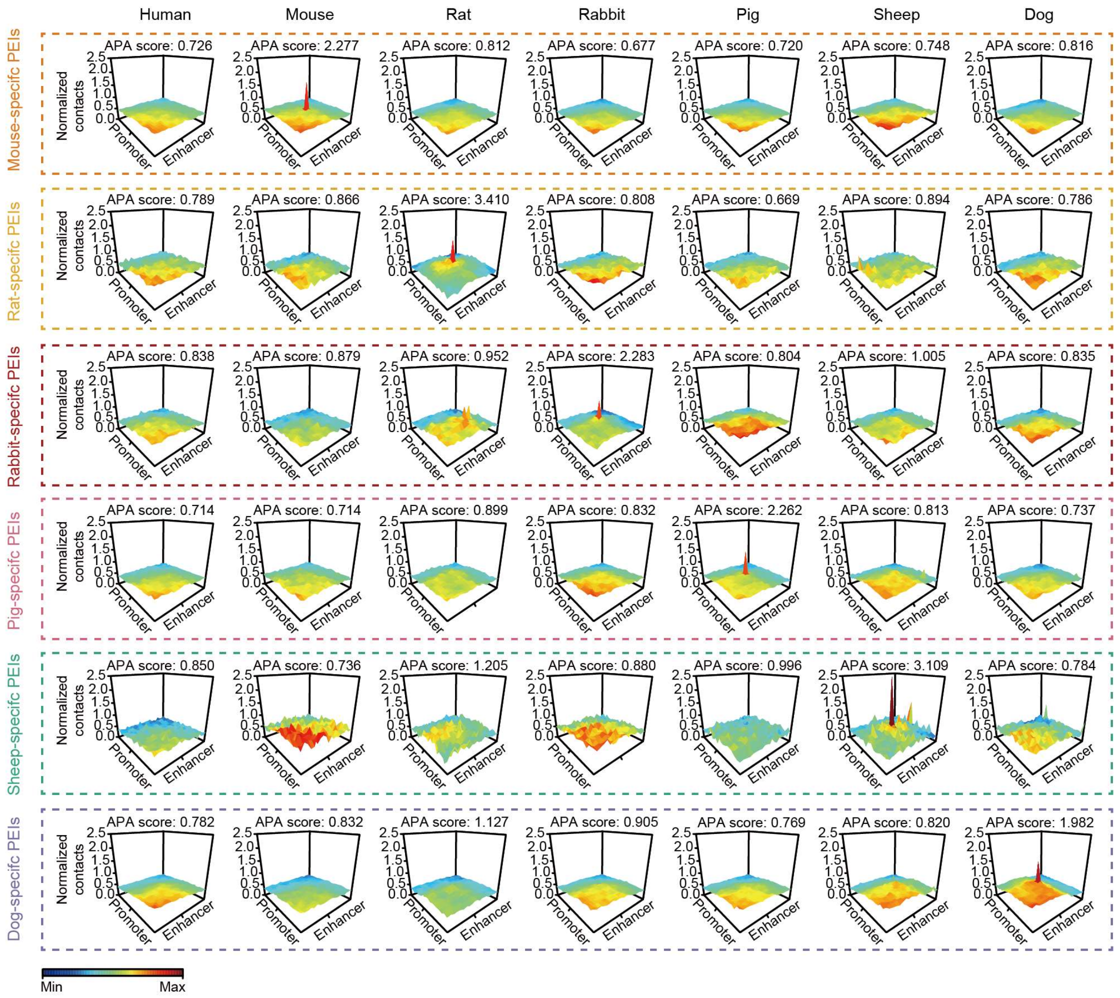

Figure. S8 Normalized Hi-C signals around species-specific PEIs in each mammalian SAT at 10 kb resolution.

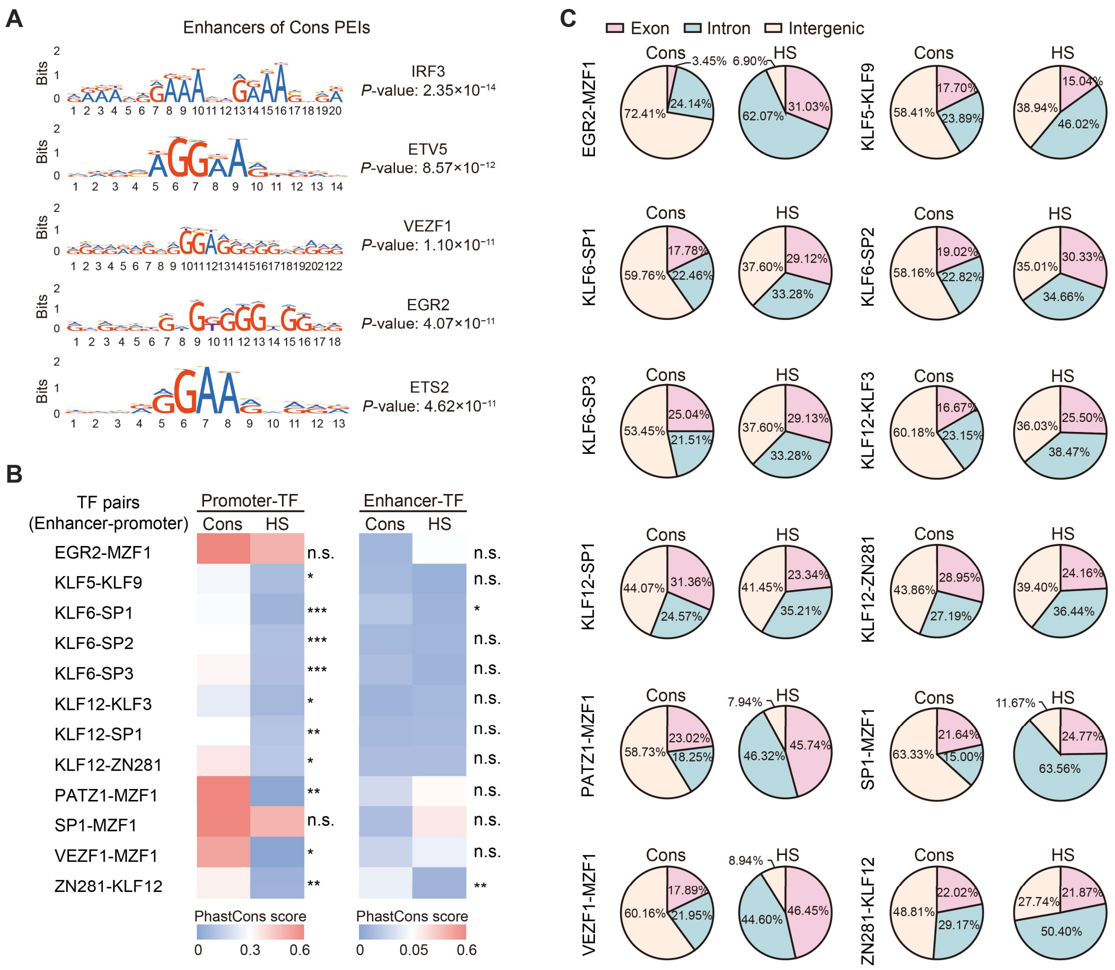

Figure. S9 Characterization of transcription factor motifs enriched in human-specific and species-conserved PEIs.

(A) Top five enriched TF motifs within the enhancer regions of Cons PEIs. **(B)** Sequence conservation of the binding sites of the candidate HS PEIs preferred TF pairs in Cons and HS PEIs. The base (nucleotide resolution) phastCons scores were collected from the UCSC Genome Browser based on Multiz alignment of 30 mammal species. Significance is indicated (Wilcoxon rank-sum test). n.s., $P \geq 0.05$; *, $P < 0.05$; **, $P < 0.01$; ***, $P < 0.001$. **(C)** Distribution of the enhancer-TF binding sites of HS PEIs preferred TF pairs in Cons and HS PEIs relative to human genomic features. The binding sites are divided into three categories based on over 50% overlap with features, including exon, intron, and intergenic.

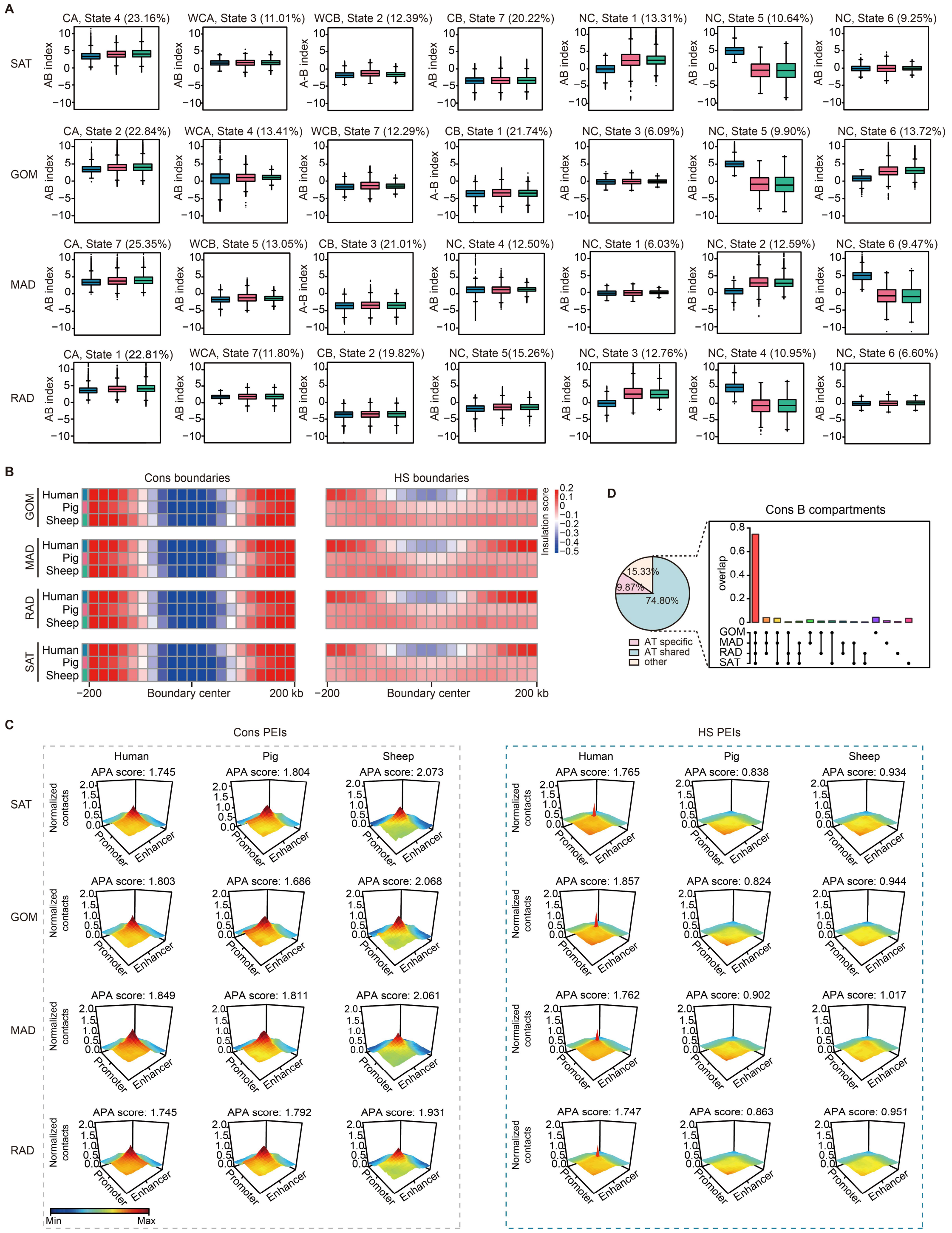

Figure. S10 Identified human-specific and species-conserved chromatin structures among humans, pigs, and sheep for each adipose tissue.

(A) Different patterns of A-B index (20 kb resolution) across three mammals predicted by Phylo-HMGP for each AT. **(B)** Average insulation score profiles in 200-kb regions centered on Cons and HS boundaries for each AT at 20 kb resolution. **(C)** Normalized Hi-C signals around Cons and HS PEIs for each AT at 10 kb resolution. **(D)** Overlap of identified Cons B compartments in four ATs.

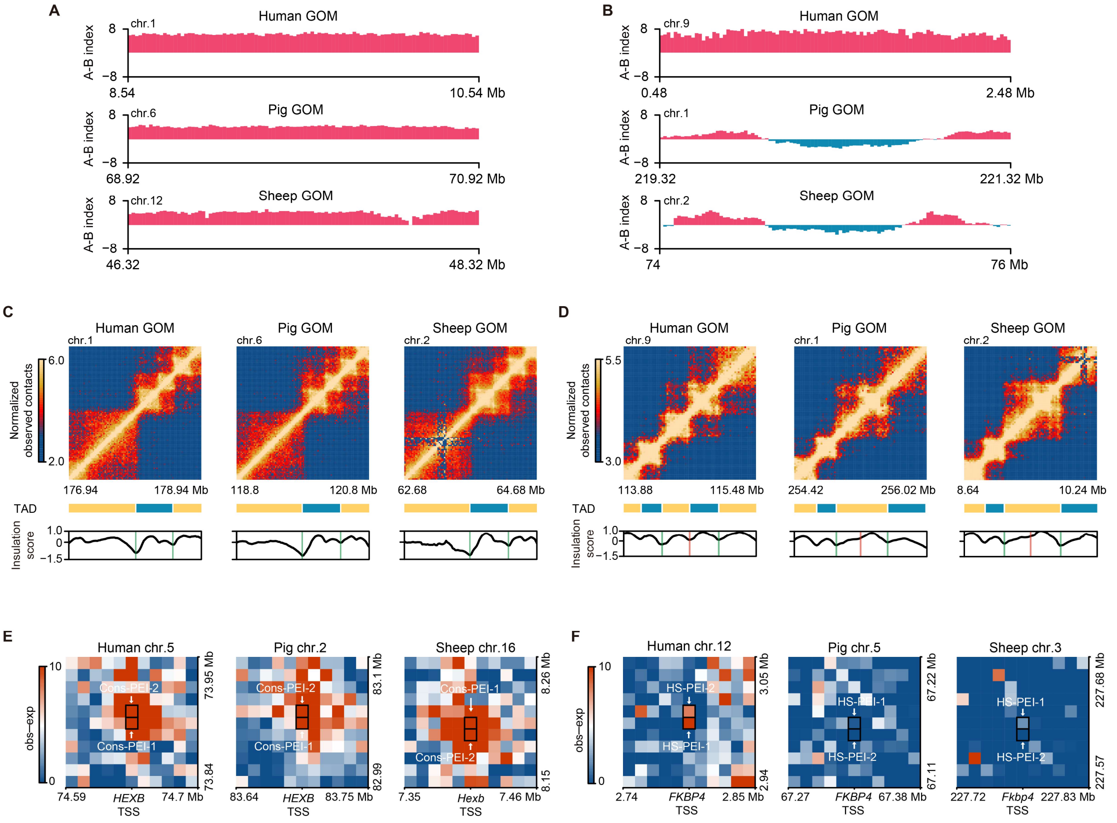

Figure. S11 Examples of human-specific and species-conserved chromatin structures among human, pig and sheep GOM.

(A-B) Cons **(A)** and HS-active **(B)** compartmentalization are indicated by the AB-index. **(C-D)** Hi-C contact maps and insulation score profiles around Cons **(C)** and HS **(D)** TADs. The Cons and HS TAD boundaries are highlighted with green and red bars, respectively, in insulation score profiles. **(E-F)** Local contact maps around Cons **(E)** and HS **(F)** PEIs (indicated by black squares).

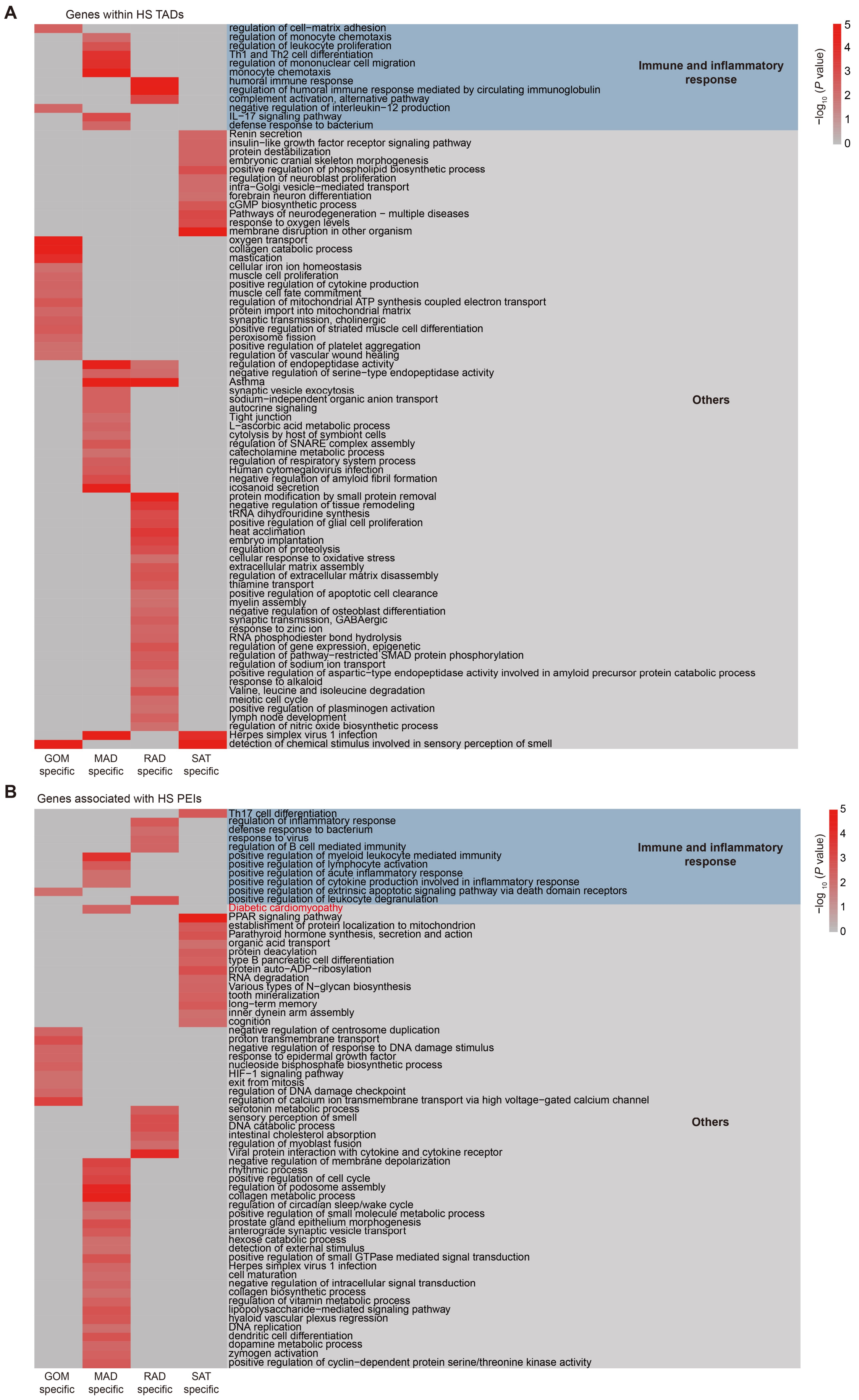
A
Genes within HS TADs
regulation of cell−matrix adhesion
regulation of monocyte chemotaxis
regulation of leukocyte proliferation
Th1 and Th2 cell differentiation
regulation of mononuclear cell migration
monocyte chemotaxis
humoral immune response
regulation of humoral immune response mediated by circulating immunoglobulin
complement activation, alternative pathway
negative regulation of interleukin−12 production
IL−17 signaling pathway
defense response to bacterium
Renin secretion
insulin−like growth factor receptor signaling pathway
protein destabilization
embryonic cranial skeleton morphogenesis
positive regulation of phospholipid biosynthetic process
regulation of neuroblast proliferation
intra−Golgi vesicle−mediated transport
forebrain neuron differentiation
cGMP biosynthetic process
Pathways of neurodegeneration − multiple diseases
response to oxygen levels
membrane disruption in other organism
oxygen transport
collagen catabolic process
mastication
cellular iron ion homeostasis
muscle cell proliferation
positive regulation of cytokine production
muscle cell fate commitment
regulation of mitochondrial ATP synthesis coupled electron transport
protein import into mitochondrial matrix
synaptic transmission, cholinergic
positive regulation of striated muscle cell differentiation
peroxisome fission
positive regulation of platelet aggregation
regulation of vascular wound healing
regulation of endopeptidase activity
negative regulation of serine−type endopeptidase activity
Asthma
synaptic vesicle exocytosis
sodium−independent organic anion transport
autocrine signaling
Tight junction
L−ascorbic acid metabolic process
cytolysis by host of symbiont cells
regulation of SNARE complex assembly
catecholamine metabolic process
regulation of respiratory system process
Human cytomegalovirus infection
negative regulation of amyloid fibril formation
icosanoid secretion
protein modification by small protein removal
negative regulation of tissue remodeling
tRNA dihydrouridine synthesis
positive regulation of glial cell proliferation
heat acclimation
embryo implantation
regulation of proteolysis
cellular response to oxidative stress
extracellular matrix assembly
regulation of extracellular matrix disassembly
thiamine transport
positive regulation of apoptotic cell clearance
myelin assembly
negative regulation of osteoblast differentiation
synaptic transmission, GABAergic
response to zinc ion
RNA phosphodiester bond hydrolysis
regulation of gene expression, epigenetic
regulation of pathway−restricted SMAD protein phosphorylation
regulation of sodium ion transport
positive regulation of aspartic−type endopeptidase activity involved in amyloid precursor protein catabolic process
response to alkaloid
Valine, leucine and isoleucine degradation
meiotic cell cycle
positive regulation of plasminogen activation
lymph node development
regulation of nitric oxide biosynthetic process
Herpes simplex virus 1 infection
detection of chemical stimulus involved in sensory perception of smell
Immune and inflammatory response
Others
GOM specific
MAD specific
RAD specific
SAT specific
−log10 (P value)
5
4
3
2
1
0
B
Genes associated with HS PEIs
Th17 cell differentiation
regulation of inflammatory response
defense response to bacterium
response to virus
regulation of B cell mediated immunity
positive regulation of myeloid leukocyte mediated immunity
positive regulation of lymphocyte activation
positive regulation of acute inflammatory response
positive regulation of cytokine production involved in inflammatory response
positive regulation of extrinsic apoptotic signaling pathway via death domain receptors
positive regulation of leukocyte degranulation
Diabetic cardiomyopathy
PPAR signaling pathway
establishment of protein localization to mitochondrion
Parathyroid hormone synthesis, secretion and action
organic acid transport
protein deacylation
type B pancreatic cell differentiation
protein auto−ADP−ribosylation
RNA degradation
Various types of N−glycan biosynthesis
tooth mineralization
long−term memory
inner dynein arm assembly
cognition
negative regulation of centrosome duplication
proton transmembrane transport
negative regulation of response to DNA damage stimulus
response to epidermal growth factor
nucleoside bisphosphate biosynthetic process
HIF−1 signaling pathway
exit from mitosis
regulation of DNA damage checkpoint
regulation of calcium ion transmembrane transport via high voltage−gated calcium channel
serotonin metabolic process
sensory perception of smell
DNA catabolic process
intestinal cholesterol absorption
regulation of myoblast fusion
Viral protein interaction with cytokine and cytokine receptor
negative regulation of membrane depolarization
rhythmic process
positive regulation of cell cycle
regulation of podosome assembly
collagen metabolic process
regulation of circadian sleep/wake cycle
positive regulation of small molecule metabolic process
prostate gland epithelium morphogenesis
anterograde synaptic vesicle transport
hexose catabolic process
detection of external stimulus
positive regulation of small GTPase mediated signal transduction
Herpes simplex virus 1 infection
cell maturation
negative regulation of intracellular signal transduction
collagen biosynthetic process
regulation of vitamin metabolic process
lipopolysaccharide−mediated signaling pathway
hyaloid vascular plexus regression
DNA replication
dendritic cell differentiation
dopamine metabolic process
zymogen activation
positive regulation of cyclin−dependent protein serine/threonine kinase activity
Immune and inflammatory response
Others
GOM specific
MAD specific
RAD specific
SAT specific
−log10 (P value)
5
4
3
2
1
0

Figure. S12 Function of genes associated with AT-specific human-specific TADs (A) and human-specific PEIs (B).

The color shades on the Y-axes highlight the categories of GO terms: immune and inflammatory response related terms (blue) and others (grey).
